# SO_2_F_2_-Mediated one-pot cascade process for transformation of aldehydes (RCHO) to cyanamides (RNHCN)†

**DOI:** 10.1039/d0ra02631j

**Published:** 2020-05-04

**Authors:** Yiyong Zhao, Junjie Wei, Shuting Ge, Guofu Zhang, Chengrong Ding

**Affiliations:** College of Chemical Engineering, Zhejiang University of Technology Hangzhou 310014 People's Republic of China gfzhang@zjut.edu.cn dingcr@zjut.edu.cn; Zhejiang Emission Trading Center Hangzhou 310014 People's Republic of China

## Abstract

A simple, mild and practical cascade process for the direct conversion of aldehydes to cyanamides was developed featuring a wide substrate scope and great functional group tolerability. This method allows for transformations of readily available, inexpensive, and abundant aldehydes to highly valuable cyanamides in a pot, atom, and step-economical manner with a green nitrogen source. This protocol will serve as a robust tool for the installation of the cyanamide moiety in various complicated molecules.

## Abstract 2

Our gram-scale process uses abundant and inexpensive aldehydes, a clean nitrogen source, requires no additional carbon atoms, is transition-metal free, and features easy work-up and excellent functional group compatibility.

## Graphical abstract

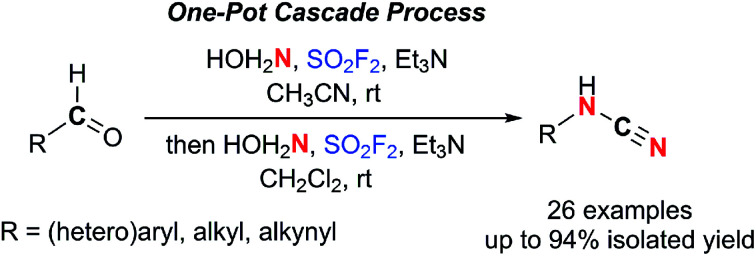

As a class of multistep, one-pot processes without the separation of intermediates, the cascade (tandem or domino) reactions have been acknowledged as one of the most powerful tools in modern chemistry with the features of atom-economy, saving power and consumption, better resource management, easy purification and lowest waste generation while still providing a higher yield than the traditional reactions.^1^ Therefore, designing controllable cascade reactions with excellent molecular efficiencies and high selectivity is a very challenging but rather highly desirable and strategic key element for modern synthetic and sustainable chemistry.^2^ Cyanamides represent the core motif in biologically active molecules and have been widely used in pharmaceuticals and functionalized materials.^3^ As a reactive N–C–N building block, cyanamides are more commonly used as a precursor in the synthesis of pharmaceutically important N-containing heterocycles and *N*-alkyl or *N*-aryl imides.^4^ Despite their versatile applications, only a limited number of synthetic routes have been reported for cyanamides in the literature.^5^ The most frequently adopted method is the direct cyanation of amines using cyanogen halides,^6^ which is overshadowed by its acute toxicity, unfavorable physical properties and sensitivity to moisture.^7^ Another straightforward approach is the direct alkylation of cyanamides; however, *N*,*N*-dialkylated cyanamides are usually obtained due to the competing alkylation of monoalkylated cyanamides.^8^ Other approaches include the dehydrosulfurization of thiourea,^9^ the dehydration of urea, and the conversion from isocyanides, isocyanates, or isothiocyanates.^10^ These above methods are mutually complementary since they all originate from the corresponding amines with multistep manipulations. In addition, some of the transformations require harsh conditions or hazardous reagents. Recently, several new cyanide sources, including CuCN,^11^ AIBN,^12^ TMSCN,^13^ and imidazolium thiocyanates,^14^ were employed in the direct *N*-cyanation of amines to synthesize cyanamides. As an alternative approach, the Tiemann rearrangement of amidoximes attracted chemists' interest in the synthesis of cyanamides.^15^ In 2014, Chien reported that benzenesulfonyl chlorides (TsCl or *o*-NsCl) promoted the Tiemann rearrangement of amidoximes to generate the corresponding cyanamides.^16^ However, it was highly dependent on the electronic effect of the substrates and required rigorous reaction conditions, pre-synthesis of substrates and redundant work-up.

Recently, sulfuryl fluoride (SO_2_F_2_),^17^ an inexpensive (about 1$ per kg), abundant and relatively inert electrophile (stable up to 400 °C when dry) has attracted significant attention to be used for SuFEx click chemistry and other versatile manipulations.^18^ A perusal of the literature revealed that the protons of phenolic hydroxyls or oximes hydroxyls can activate the exchange of the S–F bonds in SO_2_F_2_ for the S–O bonds to afford functional products, and the fluorosulfate functional group (–OSO_2_F) can be applied in a controllable and targeted manner for varied transformations.^19^ Most recently, our group reported a mild and robust method for efficiently converting aldoximes into the corresponding nitriles mediated by SO_2_F_2_/base in a green manner (Scheme 1, a).^20*a*^ Subsequently, an efficient method for the activation of the Beckmann rearrangement of ketoximes into amides or lactams utilizing SO_2_F_2_ was developed in our lab(Scheme 1, b).^20*b*^ Coincidentally, we found that SO_2_F_2_ could also promote the Tiemann rearrangement of amidoximes which were generated from corresponding nitriles to generate the corresponding cyanamides in good to excellent yields (Scheme 1, c).^20*c*^ Upon viewing the high value of cyanamide moieties, the easy availability of aldehydes, and our continuous efforts on the utilization of SO_2_F_2_ for chemical transformations of oximes (aldoximes, ketoximes, and amidoximes),^20^ we proposed a one-pot process for direct conversion of aldehydes to cyanamides through a cascade sequence following similar mechanism as our cascade nitrile synthesis process. We envisioned that in common polar solvent acetonitrile (CH_3_CN), aldehydes 1 would react with NH_2_OH to provide the aldoxime intermediate A after dehydration, and the aldoxime will further react with SO_2_F_2_ to generate the corresponding sulfonyl ester B, and with the assistance of the base, the following β-elimination of the precursor sulfonyl ester B would generate the desired carbon–nitrogen triple bonds of nitriles C. Subsequently, the nitriles are transformed to the amidoxime intermediate D reacting with NH_2_OH through a nucleophilic addition and dehydration process; then the amidoxime was deprotonated with SO_2_F_2_ under the promotion of the base to form the corresponding sulfonyl ester E, and the N–O bond cleavage occurred with concomitant R group migration over to the C–N bond to furnish the *N*-substituted cyanamides 2 (Scheme 1, d).

**Scheme 1:**
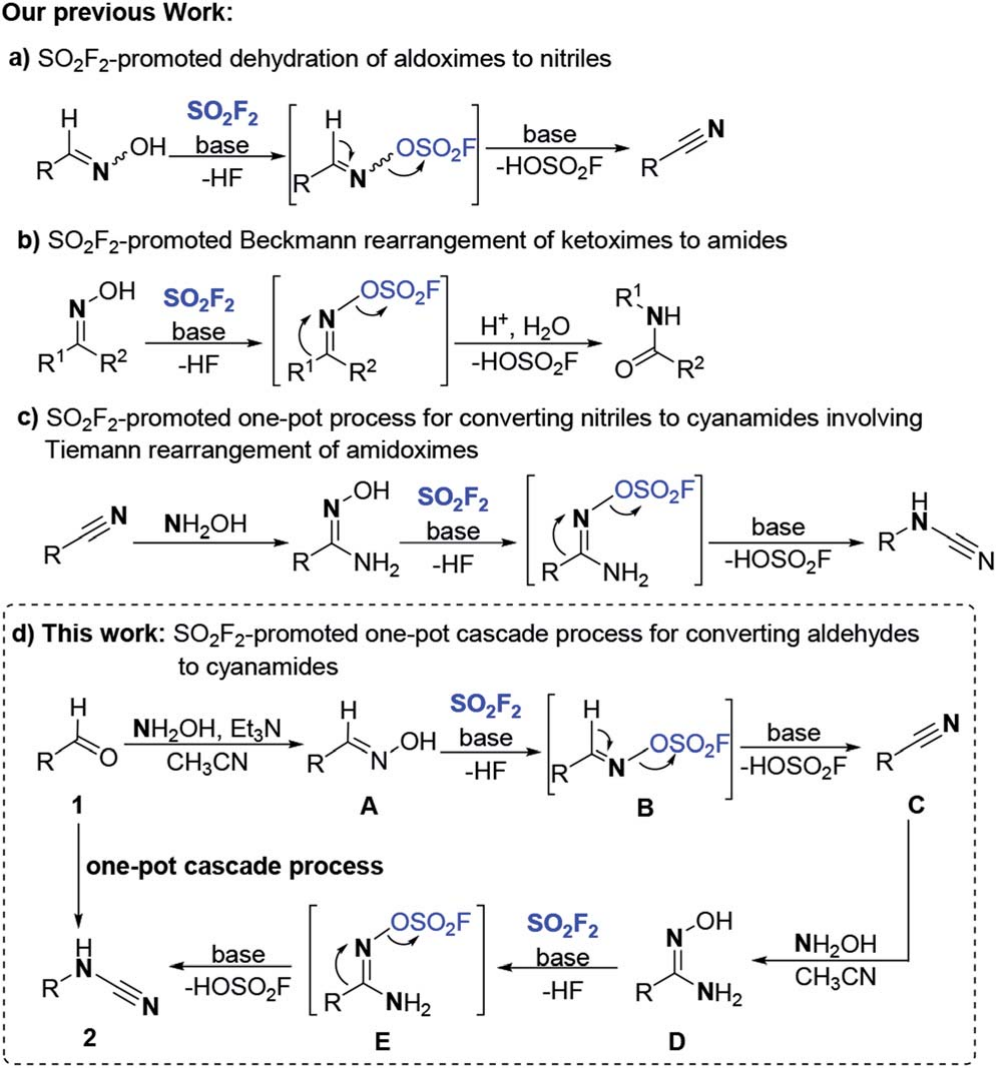
Our works on transforming of oximes mediated by SO_2_F_2_.

We conducted our initial study with benzaldehyde 1a as the model substrate to examine the feasibility of the proposed transformation. Accordingly, after screening a large variety of conditions as shown in Table 1. Considering of inorganic bases have significant advantages over their organic counterparts,^21^ inorganic bases, including K_2_CO_3_, Na_2_CO_3_, KHCO_3_, NaHCO_3_ and Na_3_PO_4_, were firstly screened (entries 1–5). Although inorganic bases were more advantageous than organic bases in Qin's oxidation system,^19^ we were disappointed to find that the use of inorganic bases provided only a trace amount of the desired product 2a. It is worth noting that the use of 1,8-diazabicyclo[5.4.0]undec-7-ene (DBU) and *N*,*N*-diisopropylethylamine (DIPEA) provided 40% and 68% isolated yields of the desired product, respectively, while the use of triethylamine (Et_3_N) assisted the reaction more efficiently to generate the desired product 2a in a great isolated yield of 94% (entries 6–8). It is not surprising to find that fixing Et_3_N as base 1 and switching base 2 to DBU and DIPEA, or fixing Et_3_N as base 2 and switching base 1 to DBU and DIPEA caused varying degrees decreased yields of the product 2a (entries 9–12).^20^ Hence, further studies were carried out by using Et_3_N as base since it provided the best yield of 2a to 94% isolated yield (entry 8). Although solid NH_2_OH·HCl is easier to operate and more inexpensive that 50 wt% NH_2_OH (aqueous solution), the moderate decreased yields of 2a were occurred when using NH_2_OH·HCl instead of 50 *wt*% NH_2_OH as the nitrogen source (entries 13, 14). Subsequently, in order to simplify the operation, the reaction mixture wasn't concentrated and carried out in CH_3_CN in the fourth step of one-pot process, however, efficiency was sharply decreased (entry 15). Therefore, the conditions of entry 8 were chosen as the standard procedure for the examinations of functional group tolerability and substrate scope.

**Table 1:** Optimization of reaction conditions^a^

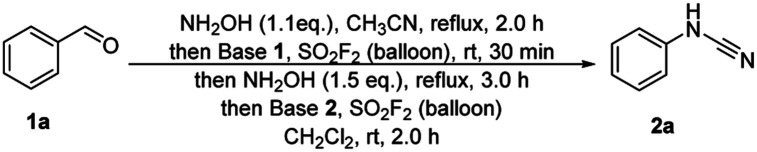			
Entry	Base 1 (2.0 equiv.)	Base 2 (2.0 equiv.)	Yield^b^ (2a, %)
1	K_2_CO_3_	K_2_CO_3_	5
2	Na_2_CO_3_	Na_2_CO_3_	<1
3	KHCO_3_	KHCO_3_	<1
4	NaHCO_3_	NaHCO_3_	<1
5	Na_3_PO_4_	Na_3_PO_4_	7
6	DBU	DBU	40
7	DIPEA	DIPEA	68
**8**	**Et**_**3**_ **N**	**Et**_**3**_ **N**	**94**
9	Et_3_N	DBU	47
10	Et_3_N	DIPEA	75
11	DBU	Et_3_N	86
12	DIPEA	Et_3_N	89
13^c^	Et_3_N	Et_3_N	90
14^d^	Et_3_N	Et_3_N	78
15^e^	Et_3_N	Et_3_N	51

a Reaction conditions: benzaldehyde 1a (1.0 mmol), 50 wt% NH_2_OH (1.2 mmol, 1.2 equiv.), CH_3_CN (10 mL), reflux, 2.0 h; then Base 1 (2.0 mmol, 2.0 equiv.), and SO_2_F_2_ balloon, r.t., 30 min; then 50 wt% NH_2_OH (1.5 mmol, 1.5 equiv.), reflux, 3.0 h; then the mixture was concentrated, base 2 (2.0 mmol, 2.0 equiv.), CH_2_Cl_2_ (10 mL), and SO_2_F_2_ balloon, r.t., 2.0 h.

b Isolated yields.

c NH_2_OH.HCl (1.2 mmol, 1.2 equiv.) and Base 1 (1.5 mmol, 1.5 equiv.) were used to replace 50 wt% NH_2_OH in the first step of one-pot process.

d NH_2_OH.HCl (1.5 mmol, 1.5 equiv.) and Base 2 (2.0 mmol, 2.0 equiv.) were used to replace 50 wt% NH_2_OH in the third step of one-pot process.

e The reaction mixture wasn't concentrated, and carried out in CH_3_CN in the fourth step of one-pot process.

Next, we evaluated the substrate scopes, functional group compatibility and limitation of the one-pot cascade process (Table 2). In most cases, the corresponding aldehydes, including aromatic and aliphatic substituted aldehydes, were successfully furnished in moderate to great yields (2a–2y). Both electron-donating (2b–2e) and electron-withdrawing groups (2f–2i) were all well tolerated under the standard reaction conditions. Notably, the satisfactory results showed that the position of the substituents on the aryl rings exhibited insignificant influence on the efficiency (2b, 2g, 2h*vs.*2k–2m*vs.*2n–2p). Furthermore, multi-functionalized benzylic aldehydes (2q, 2r) and naphthalene aldehydes (2s, 2t) were also successfully converted into their corresponding cyanamide products in moderated to great yields. Excitingly, a set of heterocyclic benzylic aldehydes (2u, 2v) were successfully converted into their corresponding cyanamides in acceptable yields. Besides, the phenylpropiolaldehyde 1w was also converted to give the final product at a 70% isolated yield (2w). For aliphatic moieties, the representative aldehydes (2x–2z) were also successfully transformed into their corresponding cyanamides with moderate yields.

**Table 2:** Scope of this SO_2_F_2_-promoted one-pot cascade process.^a^^,^^b^

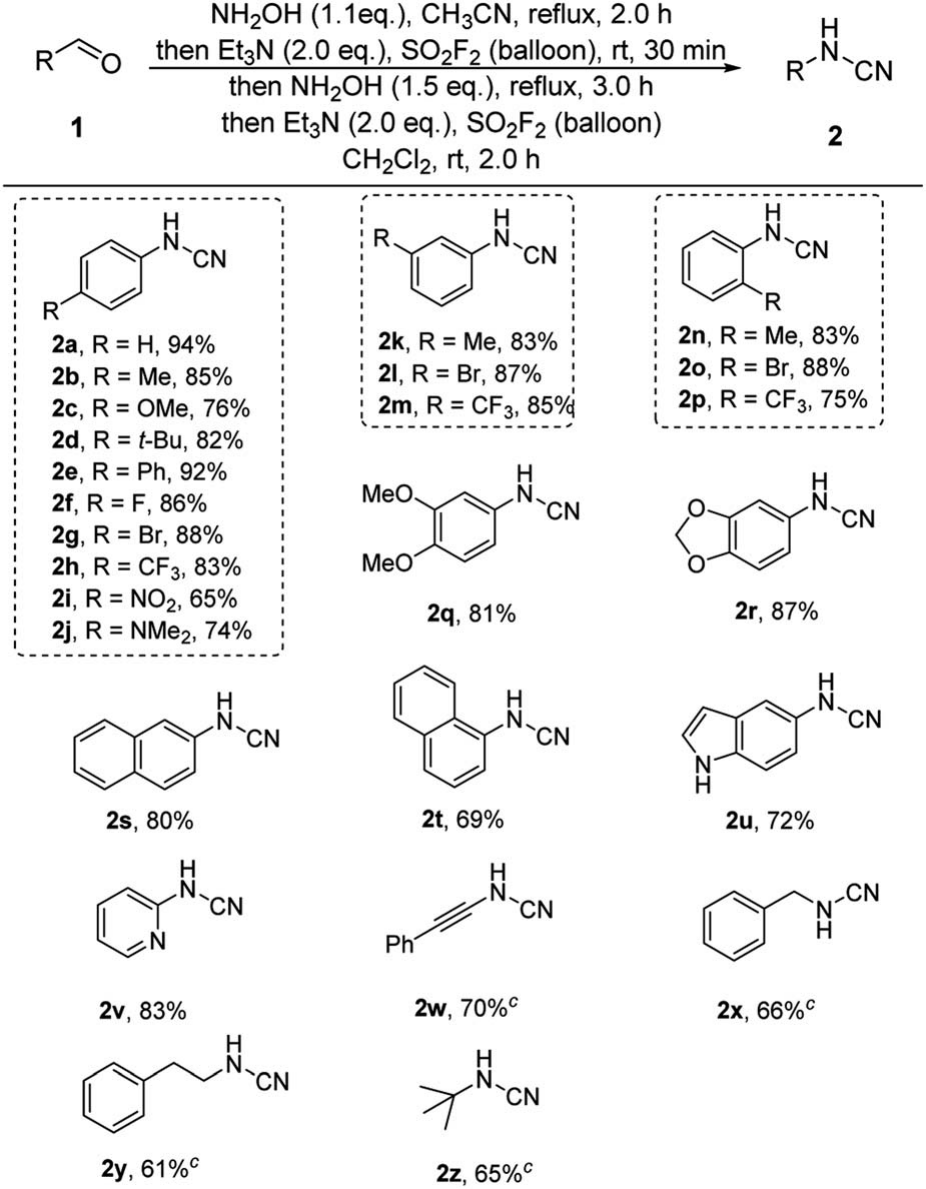

a Reaction conditions: aldehyde 1 (1.0 mmol), 50 wt% NH_2_OH (1.2 mmol, 1.2 equiv.), CH_3_CN (10 mL), reflux, 2.0 h; then Et_3_N (2.0 mmol, 2.0 equiv.), and SO_2_F_2_ balloon, r.t., 30 min; then 50 wt% NH_2_OH (1.5 mmol, 1.5 equiv.), reflux, 3.0 h; then the mixture was concentrated, Et_3_N (2.0 mmol, 2.0 equiv.), CH_2_Cl_2_ (10 mL), and SO_2_F_2_ balloon, r.t., 2.0 h.

b Isolated yields.

c Stirring 6.0 h for the fourth step of one-pot process.

In order to further demonstrate the practicality of this novel cascade process, a gram-scale (20 mmol, 2.12 g) reaction was performed under standard conditions (Scheme 2). The desired *N*-phenylcyanamide 2a was obtained with 85% isolated yield. Since the resulting product *N*-phenylcyanamide 2a is widely applied as an estimable building block in the direct and efficient synthesis of many bioactive molecules, this protocol is particularly useful. Examples include tetrazolamine 3,^22^ urea 4,^23^ amide 5,^24^ guanidine 6,^25^ 2-aminoquinazolin-4-one 7,^26^ and aminobenzonitrile 8.^27^ These representative transformations clearly demonstrate the versatility of cyanamides in organic chemistry.

**Scheme 2:**
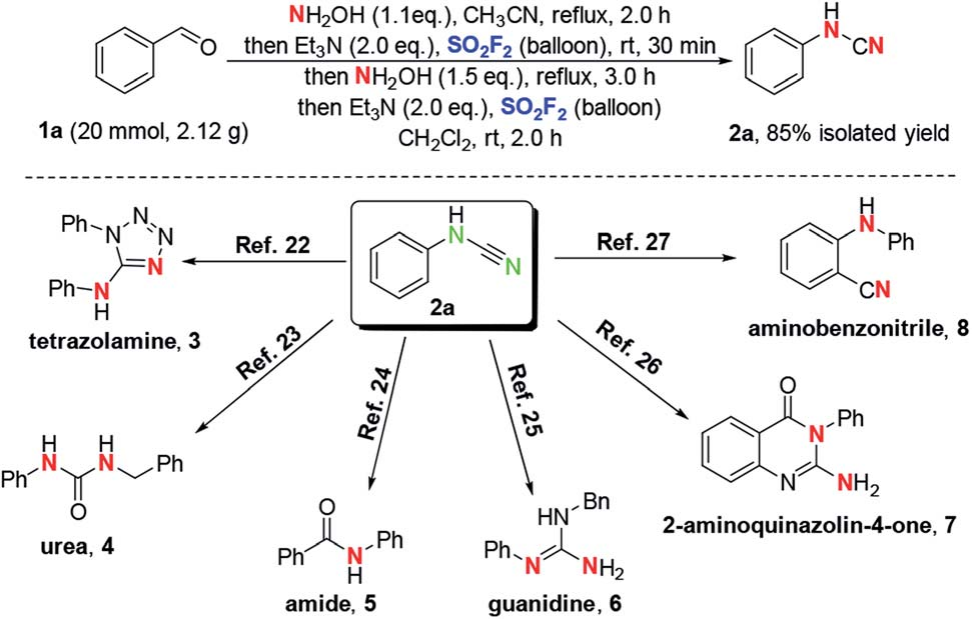
The gram-scale preparation and further transformations of *N*-phenylcyanamide 2a.

## Conclusions

In conclusion, we disclosed a new one-pot cascade process which allowed transformation of a broad range of inexpensive, easily accessible and abundant aldehydes into cyanamides with green nitrogen source. This reported SO_2_F_2_-modiated reaction proceeded with the features of mild condition, high efficiency, wide scope, and excellent functional group compatibility. Moreover, gram-scale reaction was performed to demonstrate the applicability of cyanamides, which could be efficiently converted to various structures.

## Conflicts of interest

There are no conflicts to declare.

## Supplementary Material

RA-010-D0RA02631J-s001

## References

[cit1] Anastas P., Eghbali N. (2010). Chem. Soc. Rev..

[cit2] Hayashi Y. (2016). Chem. Sci..

[cit3] (a) Ricci A. , Amino Group Chemistry From Synthesis to the Life Sciences, Wiley-VCH, Weinheim, 2008

[cit4] La Mattina J. L. (1983). J. Heterocycl. Chem..

[cit5] Prabhath M. R. R., Williams L., Bhat S. V., Sharma P. (2017). Molecules.

[cit6] Kaupp G., Schmeyers J., Boy J. (1998). Chem.–Eur. J..

[cit7] Luttrell W. E. (2009). J. Chem. Health Saf..

[cit8] Ayres J. N., Ashford M. W., Stöckl Y., Prudhomme V., Ling K. B., Platts J. A., Morrill L. C. (2017). Org. Lett..

[cit9] Van Leusen A. M., Jagt J. C. (1970). Tetrahedron Lett..

[cit10] Škoch K., Cíarová I., Štěpnicka P. (2018). Chem.–Eur. J..

[cit11] Teng F., Yu J. T., Jiang Y., Yang H., Cheng J. (2014). Chem. Commun..

[cit12] Teng F., Yu J. T., Zhou Z., Chu H., Cheng J. (2015). J. Org. Chem..

[cit13] Zhu C., Xia J. B., Chen C. (2014). Org. Lett..

[cit14] Talavera G., Peña J., Alcarazo M. (2015). J. Am. Chem. Soc..

[cit15] Tiemann F. (1891). Ber. Dtsch. Chem. Ges., Beil..

[cit16] Lin C. C., Hsieh T. H., Liao P. Y., Liao Z. Y., Chang C. W., Shih Y. C., Yeh W. H., Chien T. C. (2014). Org. Lett..

[cit17] (c) SO_2_F_2_ is commercially available from chemical vendors worldwide, http://synquestlabs.com/product/id/51619.html

[cit18] Chen W., Dong J., Plate L., Mortenson D. E., Brighty G. J., Li S., Liu Y., Galmozzi A., Lee P. S., Hulce J. J., Cravatt B. F., Saez E., Powers E. T., Wilson I. A., Sharpless K. B., Kelly J. W. (2016). J. Am. Chem. Soc..

[cit19] Hanley P. S., Clark T. P., Krasovskiy A. L., Ober M. S., O'Brien J. P., Staton T. S. (2016). ACS Catal..

[cit20] Zhao Y. Y., Mei G. Y., Wang H. B., Zhang G. F., Ding C. R. (2019). Synlett.

[cit21] Sheldon R. A. (2012). Chem. Soc. Rev..

[cit22] Boddapati S. N. M., Polam N., Mutchu B. R., Bollikolla H. B. (2018). New J. Chem..

[cit23] Basavaprabhu H., Sureshbabu V. V. (2012). Org. Biomol. Chem..

[cit24] Du J., Luo K., Zhang X. L. (2014). RSC Adv..

[cit25] Rao B., Zeng X. M. (2014). Org. Lett..

[cit26] Liao Z. Y., Yeh W. H., Liao P. Y., Liu Y. T., Chen Y. C., Chen Y. H., Hsieh T. H., Lin C. C., Lu M. H., Chen Y. S., Hsu M. C., Li T. K., Chien T. C. (2018). Org. Biomol. Chem..

[cit27] Zeng C. J., Chen C. J., Chang C. W., Chen H. T., Chien T. C. (2014). Aust. J. Chem..

