# Expression of concern: SO_2_F_2_-Mediated one-pot cascade process for transformation of aldehydes (RCHO) to cyanamides (RNHCN)

**DOI:** 10.1039/d3ra90099a

**Published:** 2023-10-11

**Authors:** Yiyong Zhao, Junjie Wei, Shuting Ge, Guofu Zhang, Chengrong Ding

**Affiliations:** a College of Chemical Engineering, Zhejiang University of Technology Hangzhou 310014 People's Republic of China gfzhang@zjut.edu.cn dingcr@zjut.edu.cn; b Zhejiang Emission Trading Center Hangzhou 310014 People's Republic of China

## Abstract

Expression of concern for ‘SO_2_F_2_-Mediated one-pot cascade process for transformation of aldehydes (RCHO) to cyanamides (RNHCN)’ by Yiyong Zhao *et al.*, *RSC Adv.*, 2020, **10**, 17288–17292, DOI: https://doi.org/10.1039/D0RA02631J.

*RSC Advances* is publishing this Expression of Concern in order to alert our readers to the fact that we are presently unable to confirm the accuracy of the information published in this article related to compound **2w**. A reader has raised concerns that the ^13^C NMR spectrum provided for compound **2w** does not fit the reported structure. The authors disagree with the reader's claims. An independent expert has reviewed the matter and has recommended that the authors must provide further evidence, from X-ray or other confirmatory tool, in order to prove the proposed structure of compound **2w**.

An expression of concern will continue to be associated with this article until a conclusive outcome is reached.

Laura Fisher

Executive Editor, *RSC Advances*

4th October 2023.

## Supplementary Material

