# Supplementary material for: SO2F2-Mediated one-pot cascade process for transformation of aldehydes (RCHO) to cyanamides (RNHCN)
Source: RSC Adv. 2020 May 4;10(29):17288–92. doi: 10.1039/d0ra02631j (PMC9053412; doi:10.1039/d0ra02631j)

Electronic Supplementary Material (ESI) for RSC Advances.
This journal is © The Royal Society of Chemistry 2020

Supplementary Information

SO_2F_2-Mediated one-pot cascade process for transformation of aldehydes (RCHO) to cyanamides (RNHCN)

Yiyong Zhao,[a] Jujie Wei,[b] Shuting Ge, [a] Guofu Zhang*[a] and Chengrong Ding*[a]

[a] College of Chemical Engineering, Zhejiang University of Technology, 18 Chaowang Road, Hangzhou 310014, People's Republic of China; [b] Zhejiang Emission Trading Center, Hangzhou 310014, People's Republic of China.

E-mail: gfzhang@zjut.edu.cn; dingcr@zjut.edu.cn.

Table of Contents

I. General Information..2

II. General procedure..3~4

III. Characterization data of products cyanamide **2**...5~12

IV. Reproductions of ^{1}H NMR and ^{13}C NMR spectra..13~38

I. General Information

Reagents were purchased at commercial quality and used without further purification unless otherwise stated. All aldehydes were purchased from Aladdin reagent Co., LTD (Shanghai). Analytical thin-layer chromatography (TLC) was performed on 0.2 mm coated silica gel plates (HSGF 254) and visualized using a UV light (254 or 365 nm). Yields of the products referred to isolated yields purified by column chromatography on silica gel (300-400 mesh) produced by Qingdao Marine Chemical Factory, Qingdao (China). ^{1}H and ^{13}C NMR spectra were recorded on a Bruker Avance Ⅲ HD 500 and 126 MHz NMR spectrometer in $CDCl_3$ or DMSO-d_6, respectively. Chemical shifts δ are reported in parts per million (ppm) relative to a residual undeuterated solvent as an internal reference (^{1}H δ 7.26 for $CDCl_3$, δ 2.50 for DMSO-d_6; ^{13}C δ 77.16 for $CDCl_3$, δ 39.52 for DMSO-d_6). The following abbreviations were used to explain NMR peak multiplicities: s = singlet, d = doublet, t = triplet, q = quartet, m = multiplet, and br = broad signal. High-resolution mass spectrometry was performed on an Agilent 6210 TOF-MS equipped with an ESI source of HR-MS System ESI spectrometer. Analytical and spectral data of all those known compounds are exactly matching with the reported values.

Ⅱ. General procedure

1. General procedure of converting aldehydes **1** to corresponding cyanamides **2**

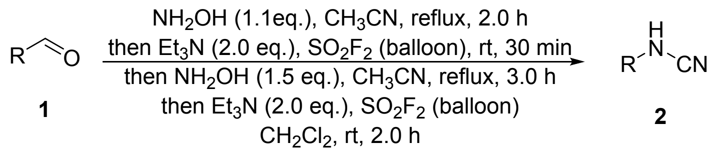

Aldehyde **1** (1.0 mmol), CH_3CN (10 mL, 0.1 M) and 50 *wt*% NH_2OH (aqueous, 79.2 mg, 1.2 mmol, 1.2 eq.) were added into a 50 mL Schlenk flask equipped with magnetic stirrer and rubber stopper, then the mixture was heated at reflux for 2.0 h. After the aldehyde was completely consumed (monitored by TLC) and cooling to room temperature, Et_3N (280 uL, 2.0 mmol, 2.0 eq.) was added, and SO_2F_2 gas was introduced into the stirring reaction mixture by slow bubbling through a SO_2F_2 balloon, and the reaction mixture was stirred at room temperature for an additional 30 min. Subsequently, another portion of 50 *wt*% NH_2OH (aqueous, 99.0 mg, 1.5 mmol, 1.5 eq.) was added into the reaction mixture, and the mixture was heated at reflux for 3.0 h. After that, the reaction mixture was concentrated under reduced pressure to remove CH_3CN, and CH_2Cl_2 (10 mL, 0.1 M), Et_3N (280 uL, 2.0 mmol, 2.0 eq.) were added into the reaction tube. Then the SO_2F_2 gas was introduced into the stirring reaction mixture by slow bubbling through a SO_2F_2 balloon, and the reaction mixture was stirred at room temperature for 2.0 h. After the reaction, the mixture was concentrated under reduced pressure and the residue was purified by column chromatography on silica gel (300-400 mesh) with hexane and ethyl acetate to give cyanamide **2**.

2. Grams-scale procedure of one-pot synthesis of *N*-phenylcyanamide **2a** from benzaldehyde **1a**

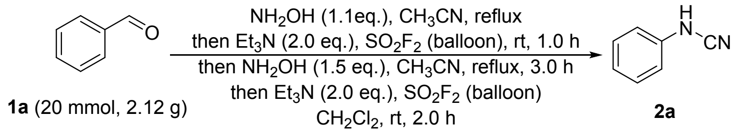

Benzaldehyde **1a** (2.12 g, 20 mmol), CH_3CN (100 mL, 0.2 M) and 50 *wt*% NH_2OH (aqueous, 1.59 g, 24 mmol, 1.2 eq.) were added into a 250 mL Schlenk flask equipped with magnetic stirrer and rubber stopper, then the mixture was heated at reflux. After benzaldehyde was completely consumed (monitored by TLC) and cooling to room temperature, Et_3N (5.6 mL, 40 mmol, 2.0 eq.) was added, and SO_2F_2 gas was introduced into the stirring reaction mixture by slow bubbling through a SO_2F_2 balloon, and the reaction mixture was stirred at room temperature for an additional 1.0 h. Subsequently, another portion of 50 *wt*% NH_2OH (aqueous, 1.98 g, 30 mmol, 1.5 eq.) was added into the reaction mixture, and the mixture was heated at reflux for 3.0 h. After that, the reaction mixture was concentrated under reduced pressure to remove CH_3CN, and CH_2Cl_2 (100 mL, 0.2 M), Et_3N (5.6 mL, 40 mmol, 2.0 eq.) were added into the reaction tube. Then the SO_2F_2 gas was introduced into the stirring reaction mixture by slow bubbling through a SO_2F_2 balloon, and the reaction mixture was stirred at room temperature for 2.0 h. After the reaction, the mixture was concentrated under reduced pressure and the residue was purified by column chromatography on silica gel (300-400 mesh) with hexane and ethyl acetate to give 2.0 g *N*-phenylcyanamide **2a**, 85% isolated yield.

Ⅲ. Characterization data of products cyanamide **2**

***N*-Phenylcyanamide (2a)**

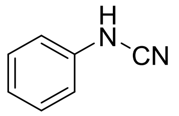

Yellow solid, 111.0 mg, 94% isolated yield. ^{1}H NMR (500 MHz, Chloroform-*d*): δ 7.34–7.28 (m, 2H), 7.08–6.88 (m, 3H); ^{13}C NMR (126 MHz, Chloroform-*d*): δ 135.84, 129.79, 128.95, 116.74, 111.09; HRMS [ESI] calcd for $C_7H_5N_2$ $[M-H]^-$: 117.0458, found: 117.0458.

***N*-(4-Methylphenyl)cyanamide (2b)**

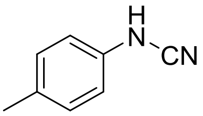

Yellow solid, 112.2 mg, 85% isolated yield. ^{1}H NMR (500 MHz, Chloroform-*d*): δ 7.15 (d, J = 8.1 Hz, 2H), 6.98–6.87 (m, 2H), 6.38 (s, 1H), 2.32 (s, 3H); ^{13}C NMR (126 MHz, Chloroform-*d*): δ 134.60, 133.32, 130.24, 115.37, 111.53, 20.64; HRMS [ESI] calcd for $C_8H_8N_2Na$ $[M+Na]^+$: 155.0580, found: 155.0586.

***N*-(4-Methoxyphenyl)cyanamide (2c)**

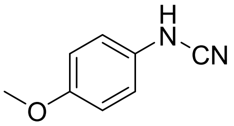

Brown solid, 112.4 mg, 76% isolated yield. ^{1}H NMR (500 MHz, Chloroform-*d*): δ 7.01–6.94 (m, 2H), 6.92–6.85 (m, 2H), 6.72 (s, 1H), 3.79 (s, 3H); ^{13}C NMR (126 MHz, Chloroform-*d*): δ 156.04, 130.34, 116.87, 115.04, 112.27, 55.64; HRMS [ESI] calcd for $C_8H_7N_2O$ $[M-H]^-$: 147.0564, found: 147.0560.

***N*-(4-*tert*-Butylphenyl)cyanamide (2d)**

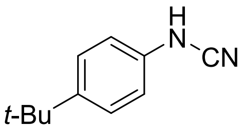

Yellow solid, 142.8 mg, 82% isolated yield. ^{1}H NMR (500 MHz, Chloroform-*d*): δ 7.42–7.33 (m, 2H), 7.07 (s, 1H), 6.99 (d, J = 8.6 Hz, 2H), 1.32 (s, 9H); ^{13}C NMR (126 MHz, Chloroform-*d*): δ 146.61, 134.66, 126.53, 115.17, 111.99, 34.26, 31.32; HRMS [ESI] calcd for $C_{11}H_{13}N_2$ [M-H]$^-$: 173.1084, found: 173.1076.

***N*-([1,1'-Biphenyl]-4-yl)cyanamide (2e)**

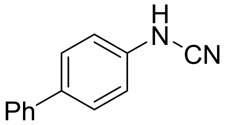

Yellow solid, 178.5 mg, 92% isolated yield. ^{1}H NMR (500 MHz, DMSO-*d₆*): δ 10.26 (s, 1H), 7.71–7.65 (m, 2H), 7.64–7.59 (m, 2H), 7.45 (t, J = 7.7 Hz, 2H), 7.33 (t, J = 7.4 Hz, 1H), 7.10–7.02 (m, 2H); ^{13}C NMR (126 MHz, DMSO- d_6): δ 139.39, 138.06, 134.52, 128.89, 127.99, 127.06, 126.19, 115.44, 111.95; HRMS [ESI] calcd for $C_{13}H_9N_2$ [M-H]$^-$: 193.0771, found: 193.0771.

***N*-(4-Fluorophenyl)cyanamide (2f)**

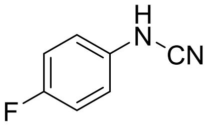

White solid, 117 mg, 86% isolated yield. ^{1}H NMR (500 MHz, Chloroform-*d*): δ 7.09–7.02 (m, 2H), 7.02–6.97 (m, 2H), 6.75 (s, 1H); ^{13}C NMR (126 MHz, Chloroform-*d*): δ 133.23, 116.95, 116.89, 116.66, 116.47, 111.45; HRMS [ESI] calcd for $C_7H_4FN_2$ [M-H]$^-$: 135.0364, found: 135.0365.

***N*-(4-Bromophenyl)cyanamide (2g)**

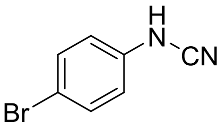

White solid, 172.5 mg, 88% isolated yield. ^{1}H NMR (500 MHz, Chloroform-*d*): δ 7.48–7.41 (m, 2H), 7.21 (s, 1H), 6.94–6.89 (m, 2H); ^{13}C NMR (126 MHz,

Chloroform-*d*): δ 136.42, 132.68, 117.12, 116.23, 111.10; HRMS [ESI] calcd for $C_7H_4BrN_2$ $[M\text{-}H]^-$: 194.9563, found: 194.9558.

N-(4-Trifluoromethylphenyl)cyanamide (2h)

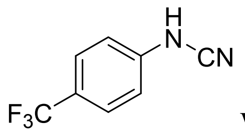

White solid, 154.4 mg, 83% isolated yield. ^{1}H NMR (500 MHz, Chloroform-*d*): δ 7.61 (d, J = 8.6 Hz, 2H), 7.31 (s, 1H), 7.13 (d, J = 8.5 Hz, 2H); ^{13}C NMR (126 MHz, Chloroform-*d*): δ 140.41, 127.21, 127.19, 127.16, 127.12, 115.41, 110.41; HRMS [ESI] calcd for $C_8H_4F_3N_2$ $[M\text{-}H]^-$: 185.0332, found: 185.0334.

N-(4-Nitrophenyl)cyanamide (2i)

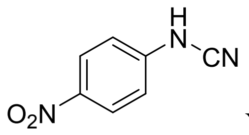

Yellow solid, 106 mg, 65% isolated yield. ^{1}H NMR (500 MHz, DMSO-d_6): δ 8.32–8.28 (m, 2H), 8.15–8.05 (m, 2H), 7.71 (s, 1H); ^{13}C NMR (126 MHz, DMSO-d_6): δ 166.16, 149.04, 139.98, 128.88, 123.38; HRMS [ESI] calcd for $C_7H_4N_3O_2$ $[M\text{-}H]^-$: 162.0309, found: 162.0310.

N-(4-Dimethylaminophenyl)cyanamide (2j)

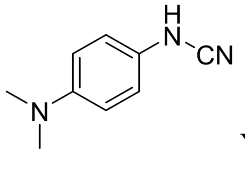

Yellow solid, 1119 mg, 74% isolated yield. ^{1}H NMR (500 MHz, DMSO-d_6): δ 6.83 (d, J = 8.9 Hz, 2H), 6.74 (d, J = 9.0 Hz, 2H), 2.83 (s, 6H); ^{13}C NMR (126 MHz, DMSO-d_6): δ 146.82, 128.19, 116.07, 113.96, 113.20, 40.63; HRMS [ESI] calcd for $C_9H_{10}N_3$ $[M\text{-}H]^-$: 160.0880, found: 160.0876.

N-(*m*-Tolyl)cyanamide (2k)

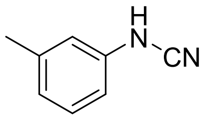

Yellow solid, 109.5 mg, 83% isolated yield. ^{1}H NMR (500 MHz, Chloroform-*d*): δ 7.22 (t, *J* = 7.8 Hz, 1H), 6.90 (d, *J* = 7.5 Hz, 1H), 6.87–6.81 (m, 2H), 2.35 (s, 3H); ^{13}C NMR (126 MHz, Chloroform- *d*): δ 139.94, 137.19, 129.51, 124.39, 115.97, 112.52, 111.67, 21.35; HRMS [ESI] calcd for $C_8H_7N_2$ [M-H]$^-$: 131.0615, found: 131.0611.

***N*-(3-Bromophenyl)cyanamide (2l)**

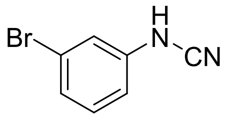

White solid, 170.5 mg, 87% isolated yield. ^{1}H NMR (500 MHz, Chloroform-*d*): δ 7.23 (q, *J* = 7.7 Hz, 3H), 6.98 (dt, *J* = 6.9, 2.1 Hz, 1H), 6.64 (s, 1H); ^{13}C NMR (126 MHz, Chloroform- *d*): δ 138.52, 131.10, 126.91, 123.46, 118.59, 114.08, 110.36; HRMS [ESI] calcd for $C_7H_4BrN_2$ [M-H]$^-$: 194.9563, found: 194.9570.

***N*-(3-Trifluoromethylphenyl)cyanamide (2m)**

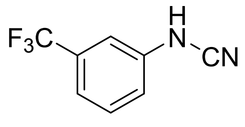

White solid, 158 mg, 85% isolated yield. ^{1}H NMR (500 MHz, Chloroform-*d*): δ 7.49 (t, *J* = 7.9 Hz, 1H), 7.44 (s, 1H), 7.36 (d, *J* = 7.8 Hz, 1H), 7.26–7.22 (m, 1H); ^{13}C NMR (126 MHz, Chloroform-*d*): δ 138.00, 132.48, 132.22, 131.96, 130.49, 124.59, 122.42, 120.48, 118.61, 112.36, 110.73; HRMS [ESI] calcd for $C_8H_4F_3N_2$ [M-H]$^-$: 185.0332, found: 185.0334.

***N*-(*o*-Tolyl)cyanamide (2n)**

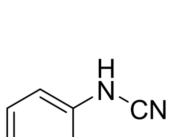

Yellow solid, 109.5 mg, 83% isolated yield. ^{1}H NMR (500 MHz, Chloroform-*d*): δ 7.29–7.20 (m, 2H), 7.17 (d, *J* = 7.5 Hz, 1H), 7.03 (td, *J* = 7.4, 1.1 Hz, 1H), 6.44 (s, 1H),

2.26 (s, 3H); ^{13}C NMR (126 MHz, Chloroform-*d*): δ 135.56, 131.00, 127.49, 124.28, 123.68, 115.46, 111.76, 17.01; HRMS [ESI] calcd for $C_8H_7N_2$ [M-H]$^-$: 131.0615, found: 131.0621.

N-(2-Bromophenyl)cyanamide (2o)

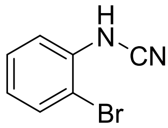

White solid, 172.5 mg, 88% isolated yield. ^1H NMR (500 MHz, Chloroform-*d*): δ 7.59–7.50 (m, 1H), 7.41–7.34 (m, 1H), 7.31 (dd, J = 8.1, 1.4 Hz, 1H), 6.99 (td, J = 7.9, 1.5 Hz, 1H), 6.51 (s, 1H); ^{13}C NMR (126 MHz, Chloroform-*d*): δ 135.22, 132.87, 131.59, 129.07, 124.74, 116.05, 109.76; HRMS [ESI] calcd for $C_7H_4BrN_2$ [M-H]$^-$: 194.9563, found: 194.9573.

N-(2-Trifluoromethylphenyl)cyanamide (2p)

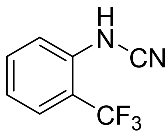

White solid, 139.5 mg, 75% isolated yield. ^1H NMR (500 MHz, Chloroform-*d*): δ 7.66–7.57 (m, 2H), 7.46 (d, J = 8.5 Hz, 1H), 7.22 (t, J = 7.7 Hz, 1H), 6.51 (s, 1H); ^{13}C NMR (126 MHz, Chloroform-*d*): δ 135.11, 133.82, 127.02, 126.98, 126.95, 126.90, 124.87, 123.62, 122.70, 117.24, 109.32; HRMS [ESI] calcd for $C_8H_4F_3N_2$ [M-H]$^-$: 185.0332, found: 185.0338.

N-(3,4-Dimethoxyphenyl)cyanamide (2q)

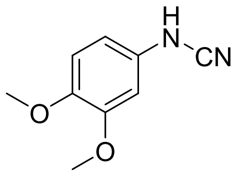

White solid, 144 mg, 81% isolated yield. ^1H NMR (500 MHz, Chloroform-*d*): δ 6.83 (d, J = 8.6 Hz, 1H), 6.61 (d, J = 2.7 Hz, 1H), 6.55 (dd, J = 8.5, 2.6 Hz, 1H), 6.10 (s, 1H), 3.89 (s, 3H), 3.87 (s, 3H); ^{13}C NMR (126 MHz, Chloroform-*d*): δ 150.15, 145.67, 130.73, 112.40, 111.57, 107.16, 100.42, 56.40, 56.09; HRMS [ESI] calcd for $C_9H_9N_2O_2$ [M-H]$^-$: 177.067, found: 177.0661.

N-(Benzo[d][1,3]dioxol-5-yl)cyanamide (2r)

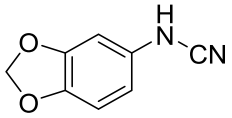

Yellow solid, 141 mg, 91% isolated yield. ^{1}H NMR (500 MHz, Chloroform-*d*): δ 6.75 (d, J = 8.3 Hz, 1H), 6.58 (d, J = 2.3 Hz, 1H), 6.53 (d, J = 16.6 Hz, 1H), 6.47 (dd, J = 8.3, 2.4 Hz, 1H), 5.97 (s, 2H); ^{13}C NMR (126 MHz, Chloroform-*d*): δ 148.71, 144.12, 131.66, 108.72, 108.05, 101.60, 98.16, 53.43; HRMS [ESI] calcd for $C_8H_5N_2O_2$ [M-H]$^-$: 161.0357, found: 161.0349.

N-(Naphthalen-2-yl)cyanamide (2s)

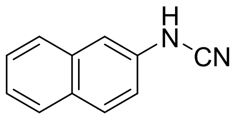

Yellow solid, 134.4 mg, 80% isolated yield. ^{1}H NMR (500 MHz, DMSO-d_6): δ 10.37 (s, 1H), 7.93 (d, J = 8.8 Hz, 1H), 7.86 (d, J = 8.9 Hz, 2H), 7.49 (ddd, J = 8.2, 6.9, 1.1 Hz, 1H), 7.43–7.36 (m, 2H), 7.20 (dd, J = 8.8, 2.4 Hz, 1H); ^{13}C NMR (126 MHz, DMSO-d_6): δ 136.42, 133.65, 129.87, 129.33, 127.69, 126.99, 126.62, 124.44, 116.27, 112.05, 110.02; HRMS [ESI] calcd for $C_{11}H_7N_2$ [M-H]$^-$: 167.0615, found: 167.0616.

N-(Naphthalen-1-yl)cyanamide (2t)

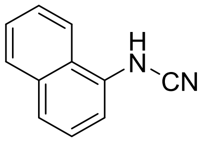

Yellow solid, 115.9 mg, 69% isolated yield. ^{1}H NMR (500 MHz, DMSO-d_6): δ 10.23 (s, 1H), 8.11–8.06 (m, 1H), 7.99–7.93 (m, 1H), 7.66 (d, J = 8.2 Hz, 1H), 7.63–7.56 (m, 2H), 7.52 (t, J = 7.9 Hz, 1H), 7.25 (d, J = 7.2 Hz, 1H); ^{13}C NMR (126 MHz, DMSO-d_6): δ 134.34, 133.86, 128.38, 126.70, 126.15, 126.01, 123.22, 123.16, 120.98, 112.63, 111.15; HRMS [ESI] calcd for $C_{11}H_7N_2$ [M-H]$^-$: 167.0615, found: 167.0615.

N-(1*H*-Indol-5-yl)cyanamide (2u)

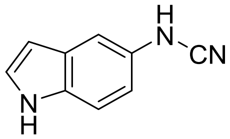

Yellow solid, 113 mg, 72% isolated yield. ^{1}H NMR (500 MHz, Chloroform-*d*): δ 8.24 (s, 1H), 7.37 (d, *J* = 8.6 Hz, 1H), 7.30 (d, *J* = 1.9 Hz, 1H), 7.26 (d, *J* = 2.7 Hz, 1H), 6.90 (dd, *J* = 8.6, 2.2 Hz, 1H), 6.51 (s, 1H), 5.80 (s, 1H); ^{13}C NMR (126 MHz, Chloroform-*d*): δ 132.88, 130.03, 128.56, 125.95, 112.16, 112.08, 111.41, 106.97, 102.41; HRMS [ESI] calcd for $C_9H_6N_3$ [M-H]$^-$: 156.0567, found: 156.0567.

***N*-(Pyridin-2-yl)cyanamide (2v)**

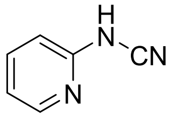

Yellow liquid, 98.8 mg, 80% isolated yield. ^{1}H NMR (500 MHz, Chloroform-*d*): δ 7.76–7.68 (m, 1H), 7.64 (ddd, *J* = 8.9, 7.0, 1.8 Hz, 1H), 7.12 (d, *J* = 8.9 Hz, 1H), 6.68–6.55 (m, 1H); ^{13}C NMR (126 MHz, Chloroform-*d*): δ 160.31, 142.12, 136.49, 118.48, 117.57, 111.56; HRMS [ESI] calcd for $C_6H_4N_3$ [M-H]$^-$: 118.0411, found: 118.0409.

***N*-(Phenylethynyl)cyanamide (2w)**

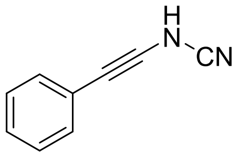

Yellow solid, 99.4 mg, 73% isolated yield. ^{1}H NMR (500 MHz, Chloroform-*d*): δ 7.72 (dd, *J* = 6.7, 3.0 Hz, 2H), 7.47–7.37 (m, 3H), 4.82 (s, 1H); ^{13}C NMR (126 MHz, Chloroform-*d*): δ 169.11, 163.82, 129.67, 128.66, 126.52, 78.02; HRMS [ESI] calcd for $C_9H_5N_2$ [M-H]$^-$: 141.0531, found: 141.0530.

***N*-Benzylcyanamide (2x)**

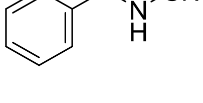

Yellow liquid, 87.1 mg, 66% isolated yield. ^{1}H NMR (500 MHz, Chloroform-*d*): δ 7.45–7.31 (m, 5H), 4.21 (d, *J* = 5.5 Hz, 2H), 4.15 (s, 1H); ^{13}C NMR (126

MHz, Chloroform-*d*): *δ* 136.18, 128.95, 128.49, 127.82, 116.01, 50.22; HRMS [ESI] calcd for $C_8H_7N_2$ [M-H]$^-$: 131.0615, found: 131.0610.

_N_-Phenethylcyanamide (2y)

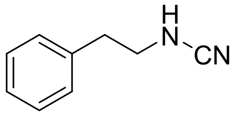

Yellow liquid, 89.2 mg, 56% isolated yield. ^{1}H NMR (500 MHz, Chloroform-*d*): *δ* 7.35 (t, *J* = 7.4 Hz, 2H), 7.30–7.25 (m, 1H), 7.22 (d, *J* = 7.1 Hz, 2H), 3.74 (s, 1H), 3.41–3.27 (m, 2H), 2.92 (t, *J* = 7.0 Hz, 2H); ^{13}C NMR (126 MHz, Chloroform-*d*): *δ* 137.21, 128.84, 128.81, 126.99, 115.87, 47.39, 35.87; HRMS [ESI] calcd for $C_9H_9N_2$ [M-H]$^-$: 145.0771, found: 145.077.

_N_-(_tert_-Butyl)cyanamide (2z)

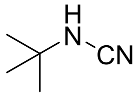

Yellow liquid, 63.8 mg, 65% isolated yield. ^{1}H NMR (500 MHz, Chloroform-*d*): *δ* 4.54 (s, 1H), 1.25 (s, 9H); ^{13}C NMR (126 MHz, Chloroform-*d*): *δ* 115.24, 53.10, 28.94; HRMS [ESI] calcd for $C_5H_{11}N_2$ [M+H]$^+$: 99.0844, found: 99.0843

Ⅳ.Reproductions of ^{1}H NMR and ^{13}C NMR spectra

***N*-phenylcyanamide (2a)**

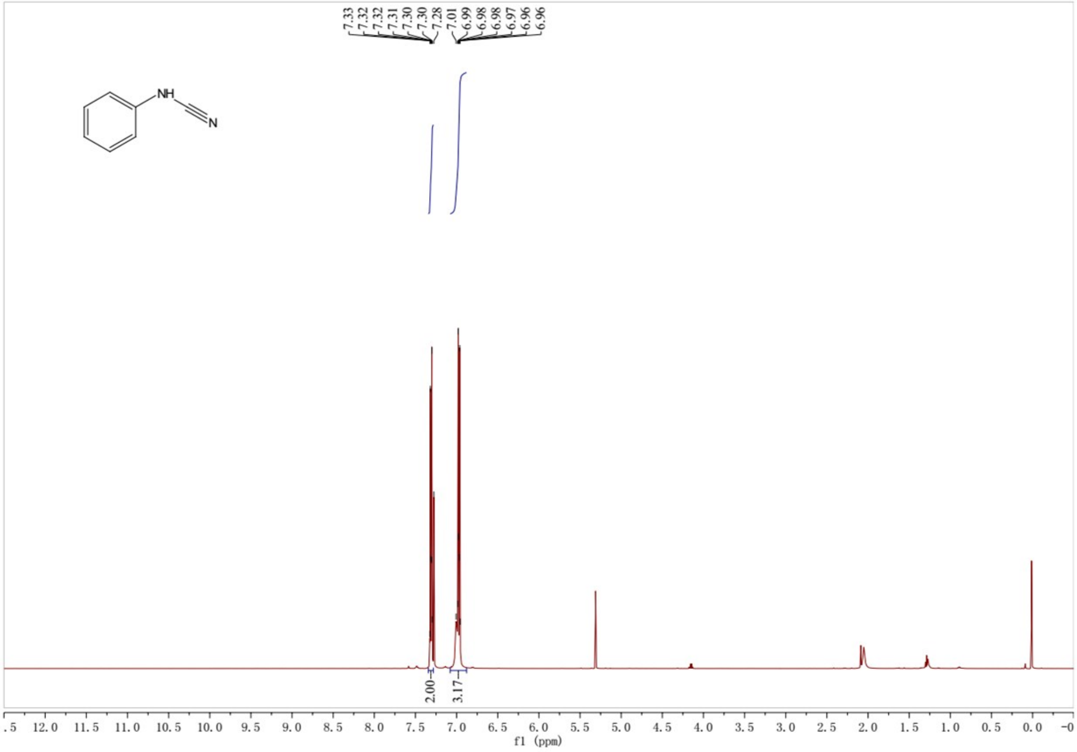

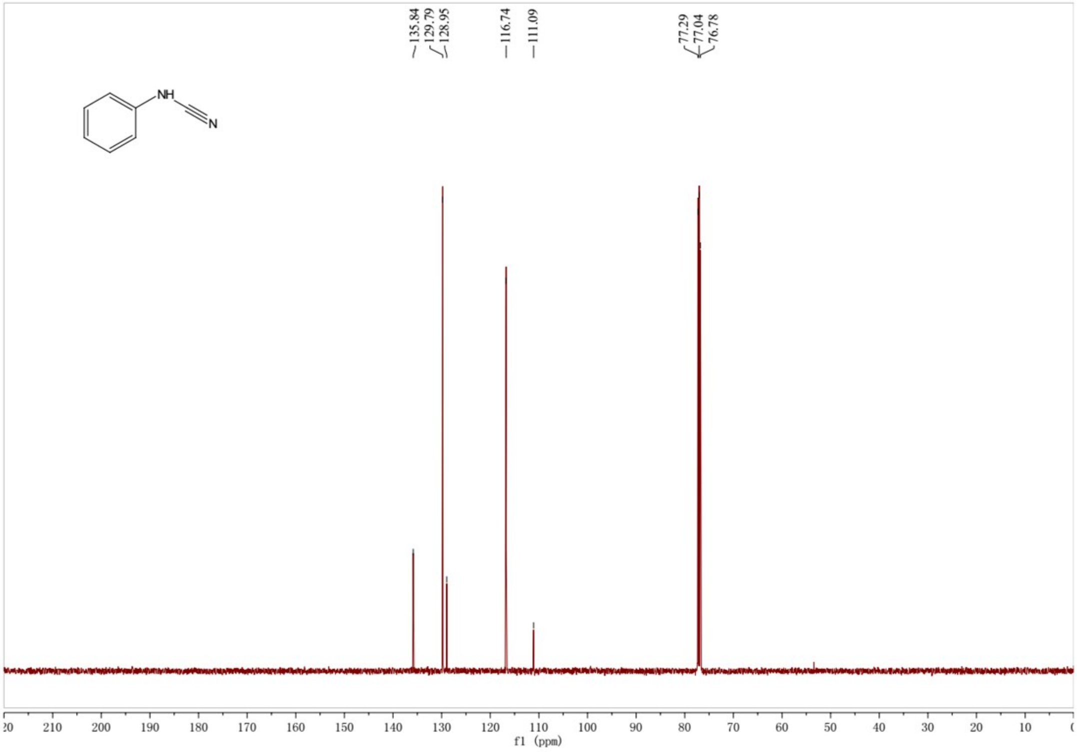

N-(4-Methylphenyl)cyanamide (2b)

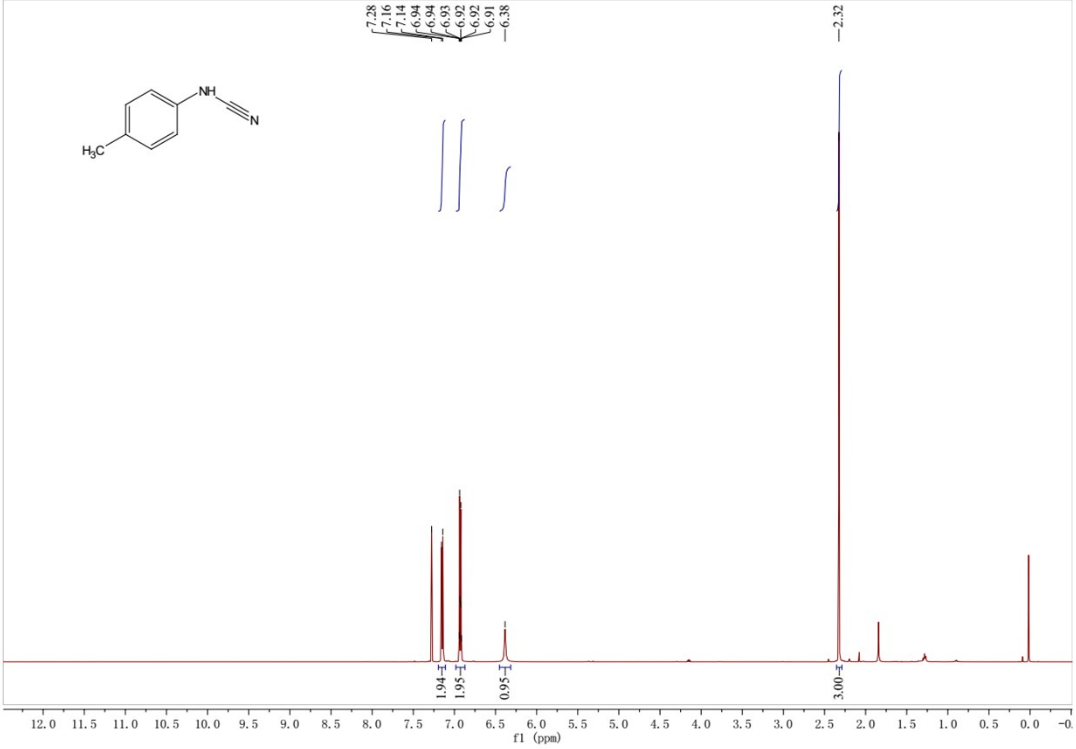

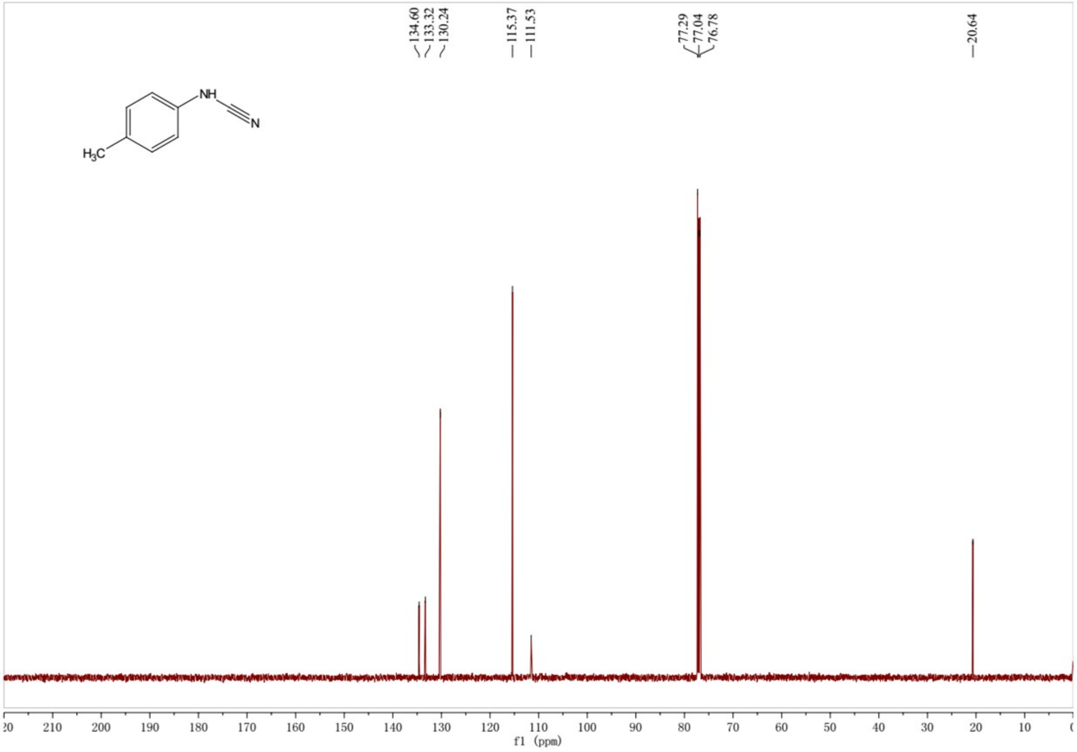

***N*-(4-Methoxyphenyl)cyanamide (2c)**

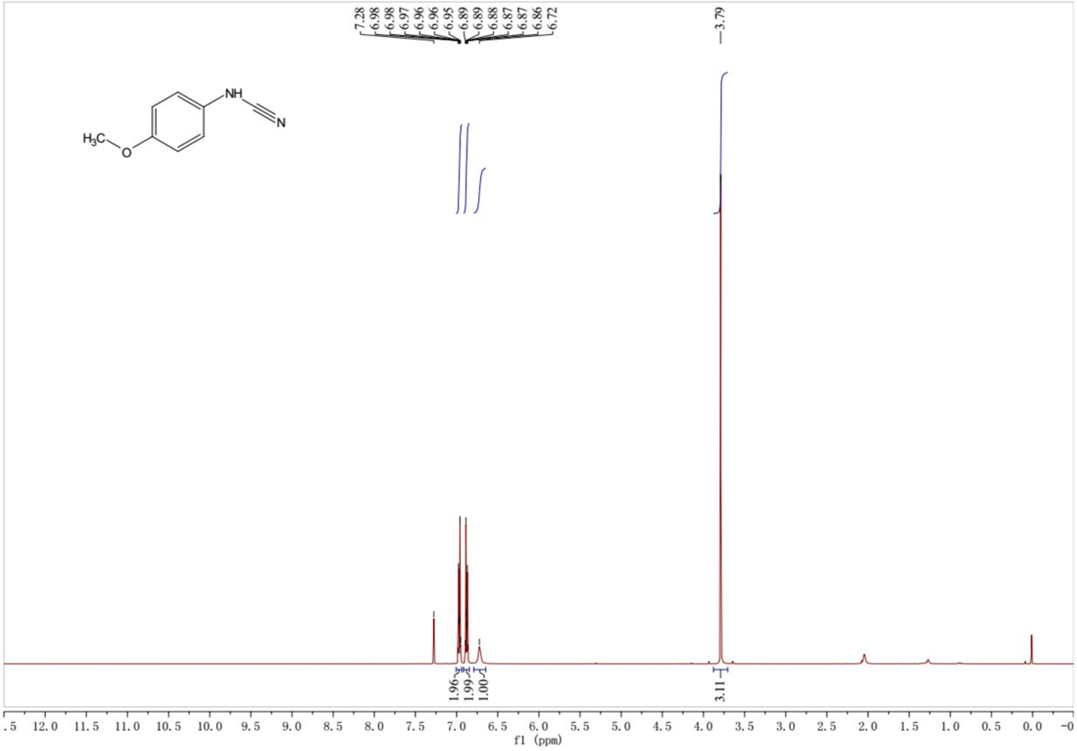

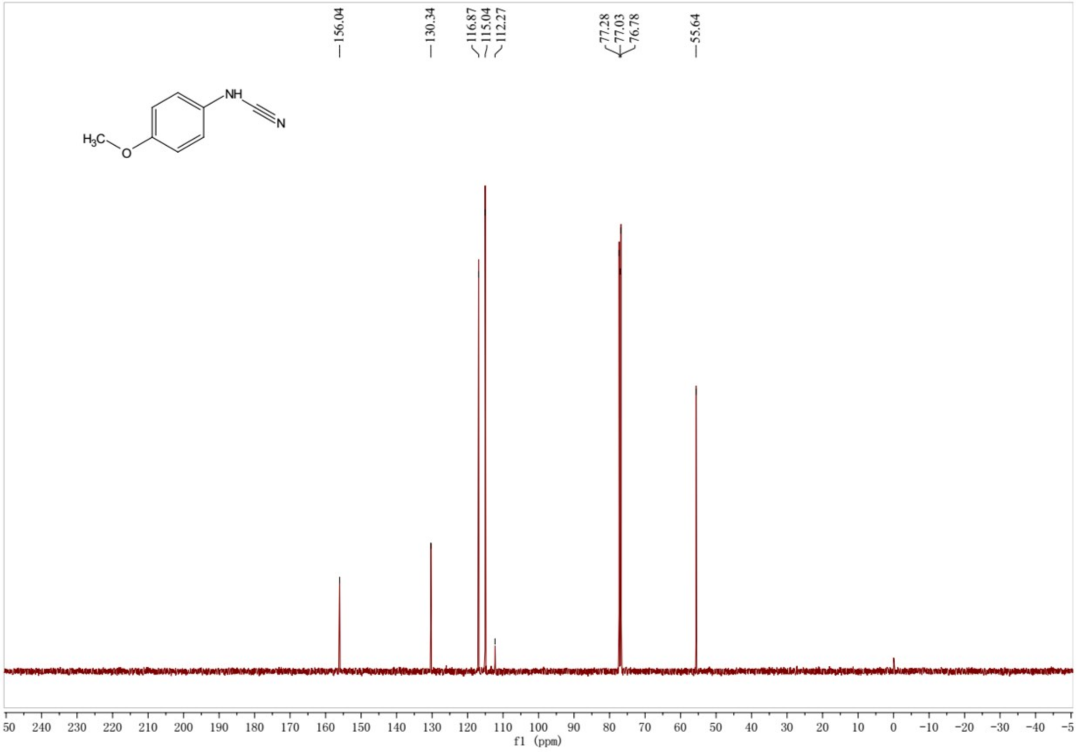

N-(4-tert-Butylphenyl)cyanamide (2d)

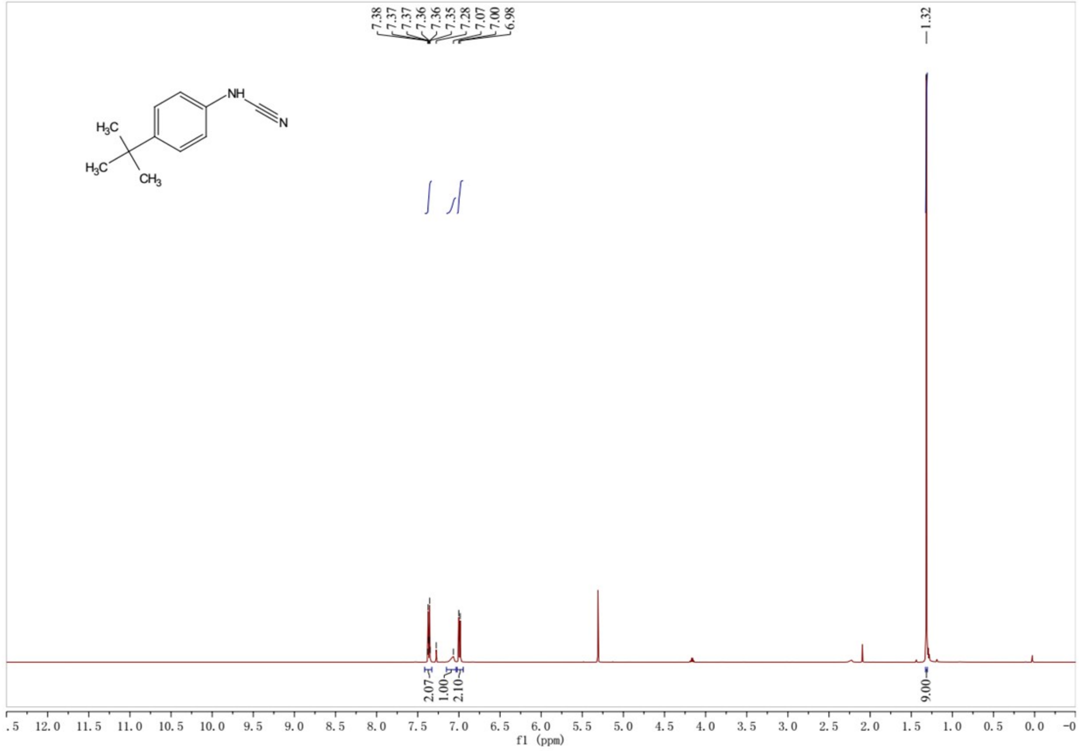

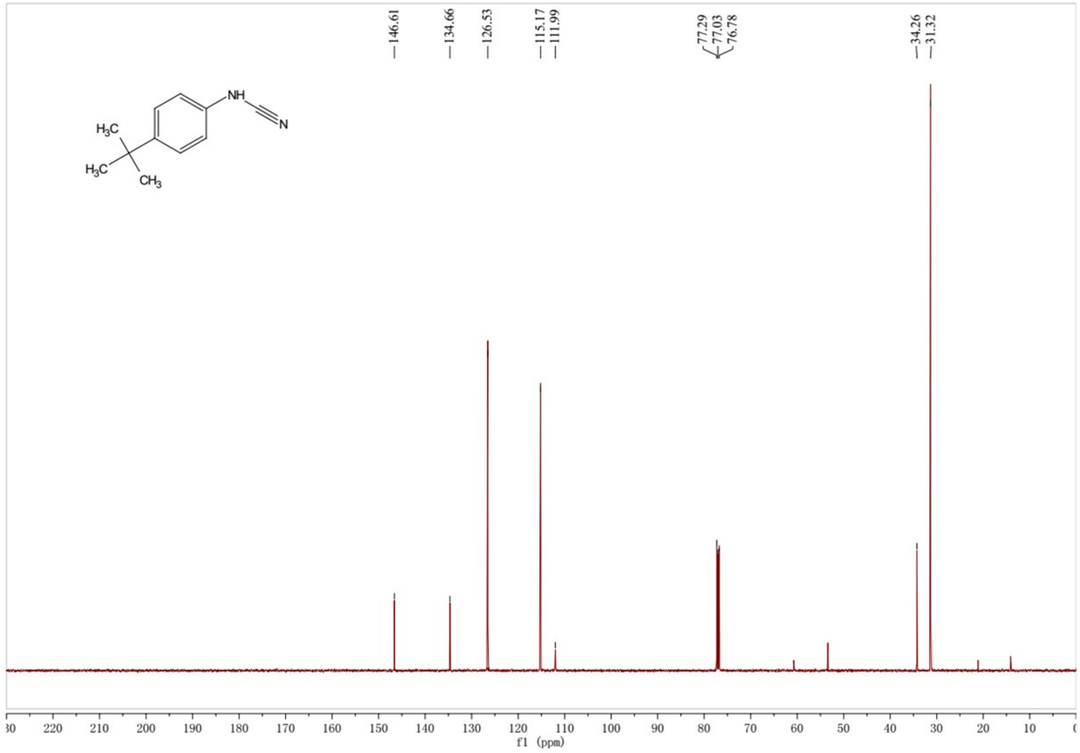

N-([1,1'-Biphenyl]-4-yl)cyanamide (2e)

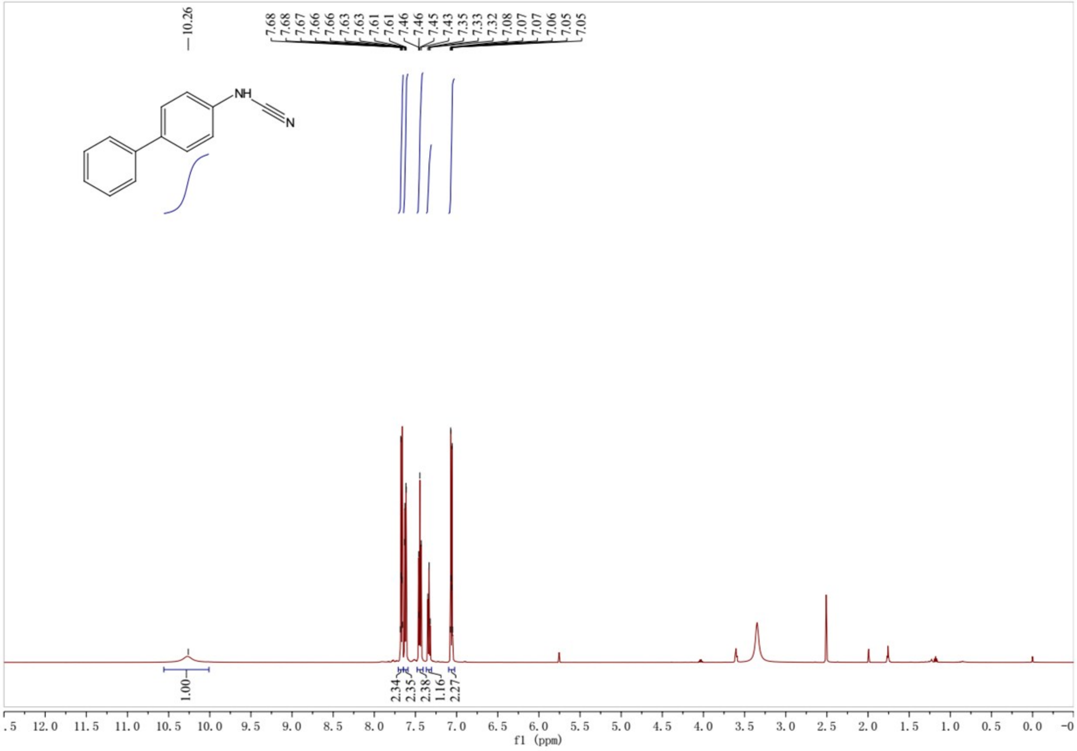

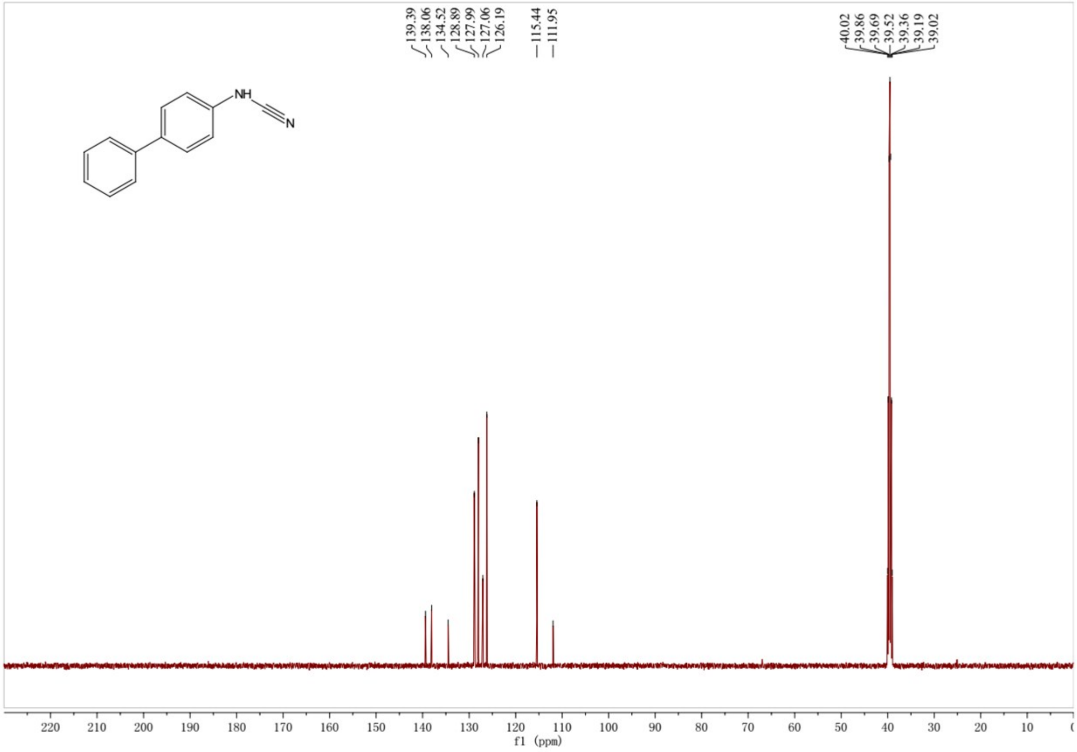

N-(4-Fluorophenyl)cyanamide (2f)

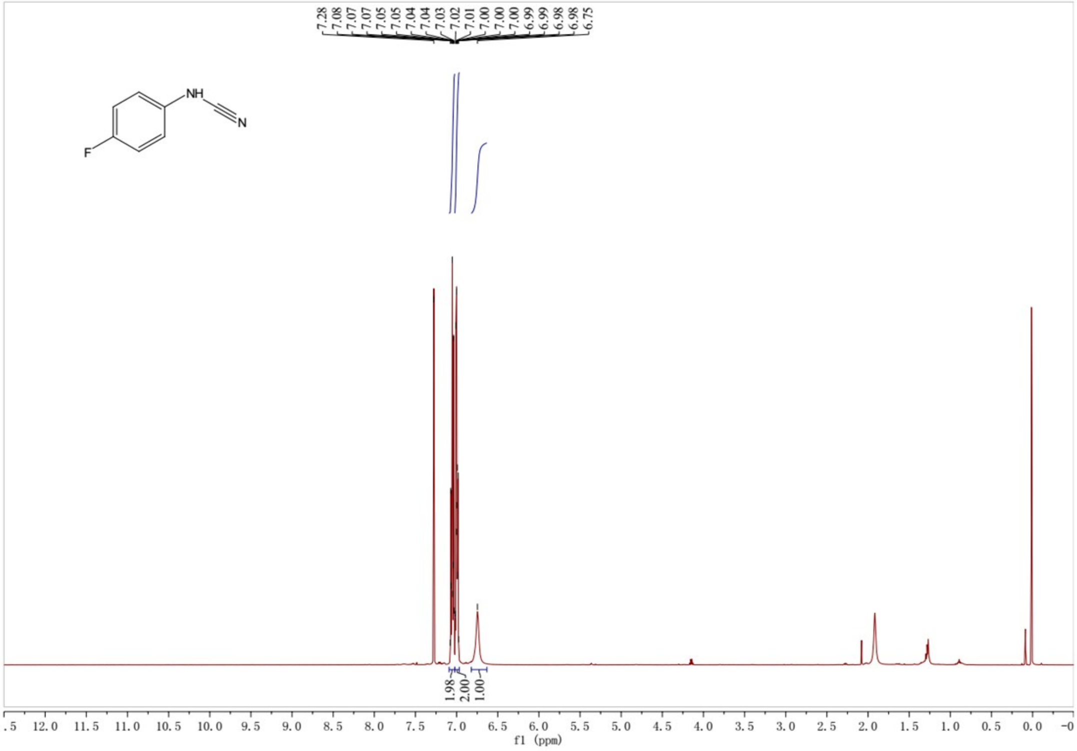

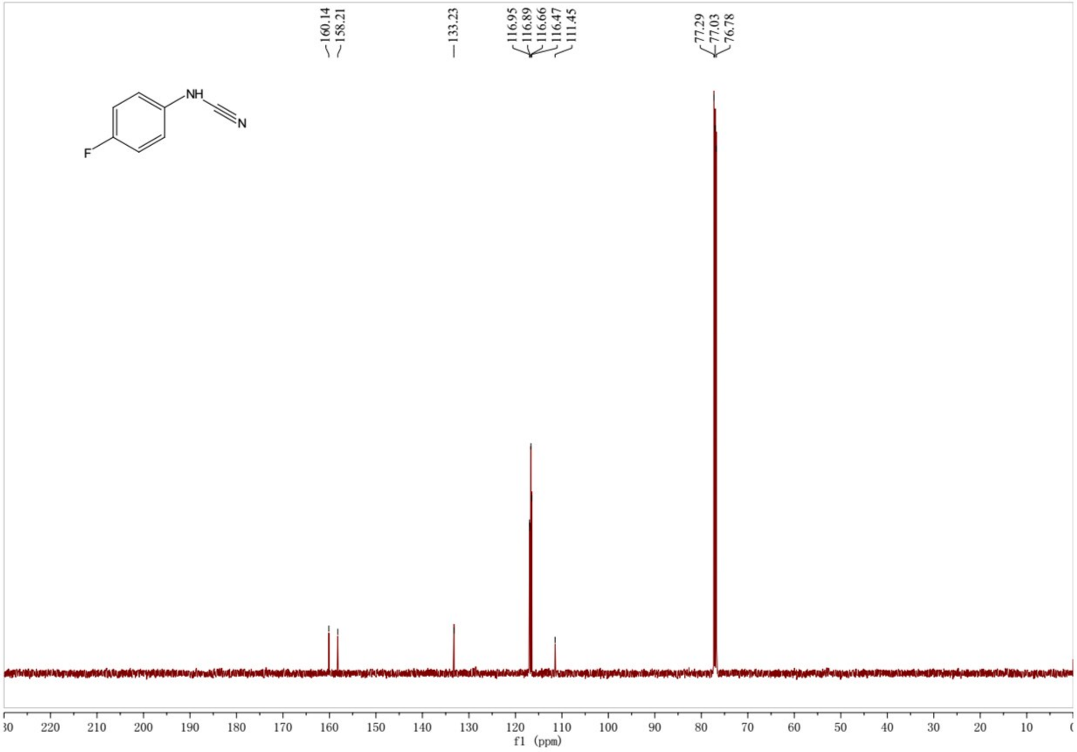

***N*-(4-Bromophenyl)cyanamide (2g)**

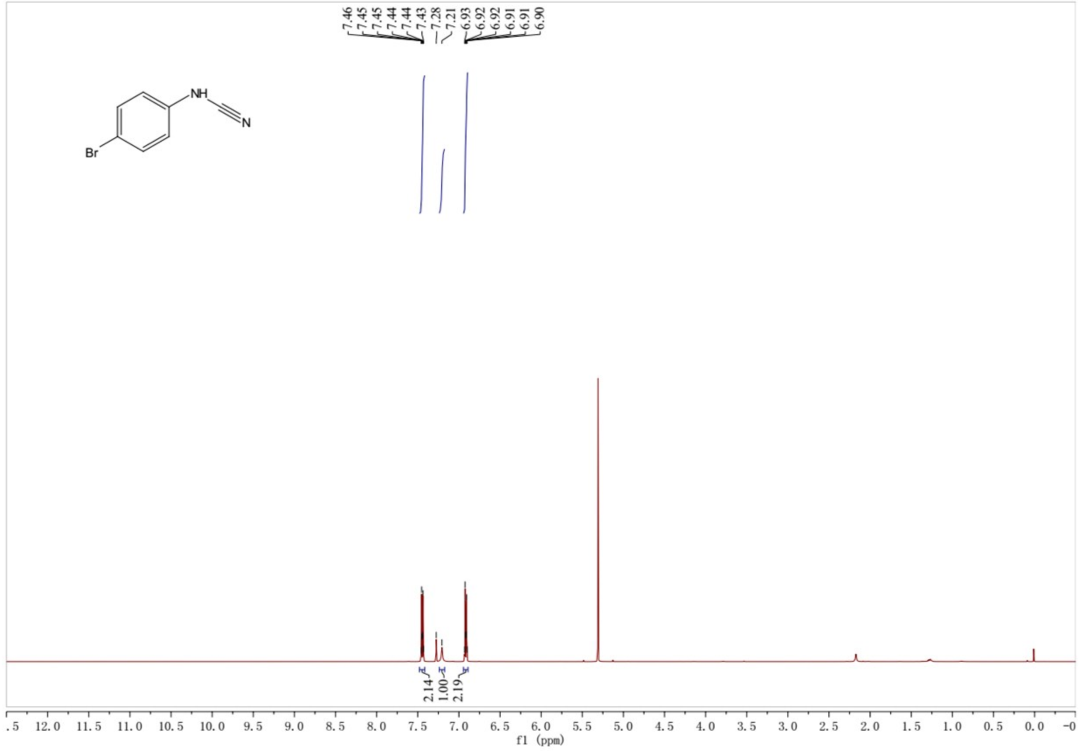

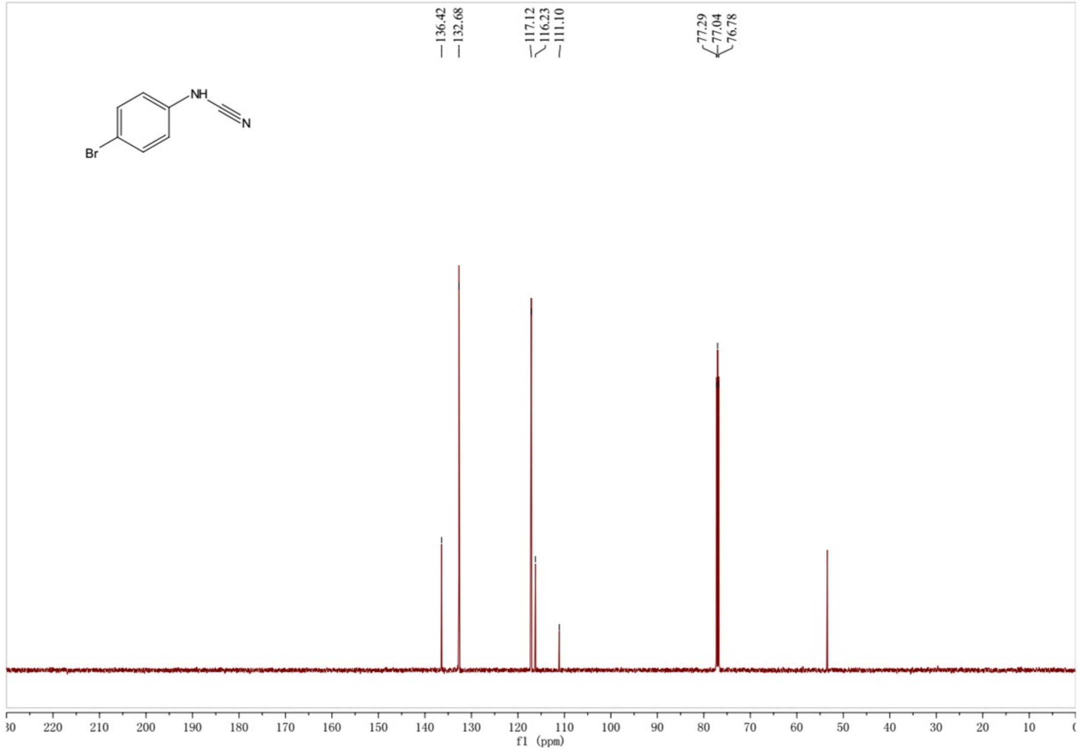

N-(4-Trifluoromethylphenyl)cyanamide (2h)

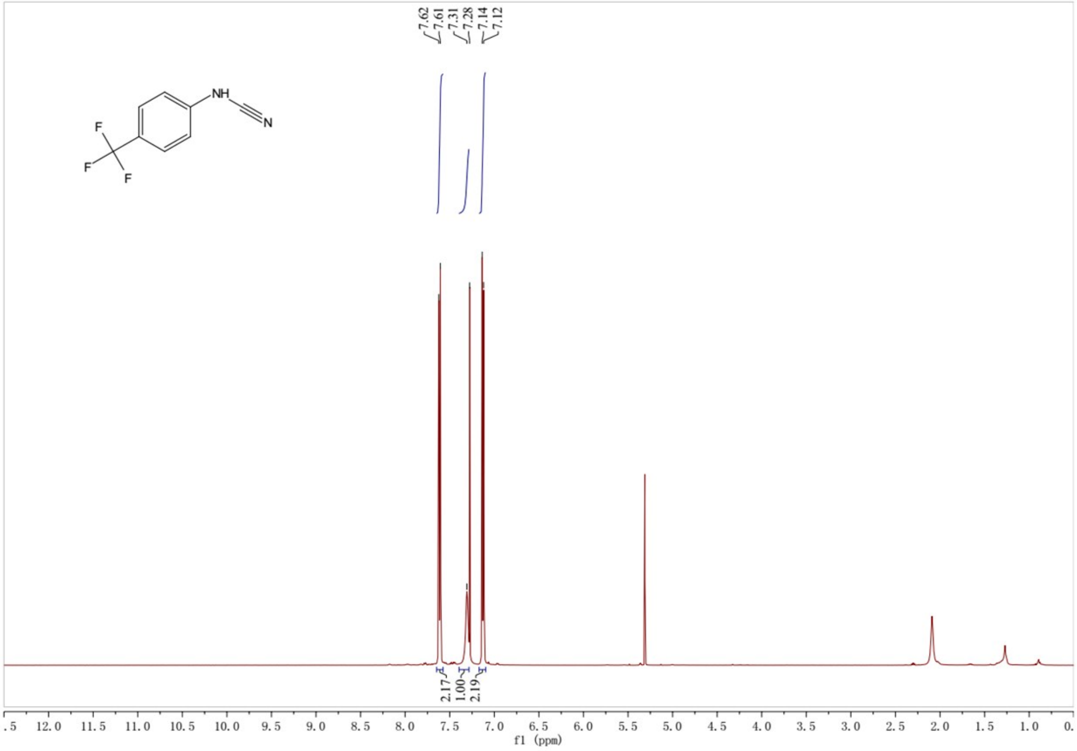

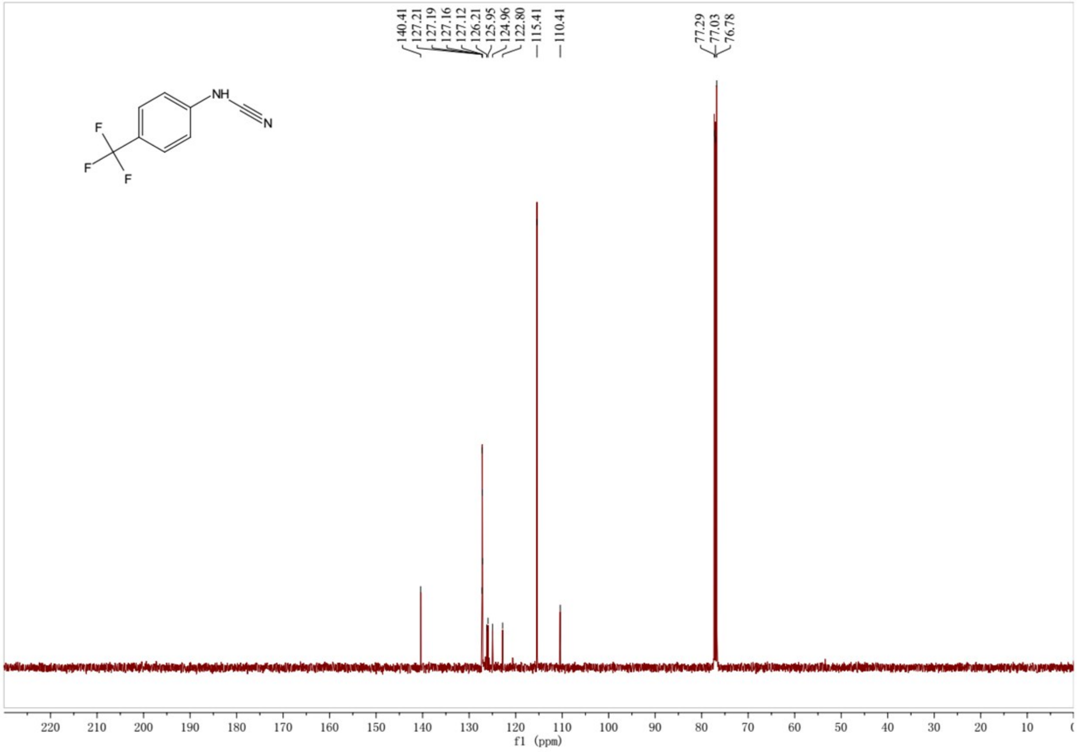

N-(4-Nitrophenyl)cyanamide (2i)

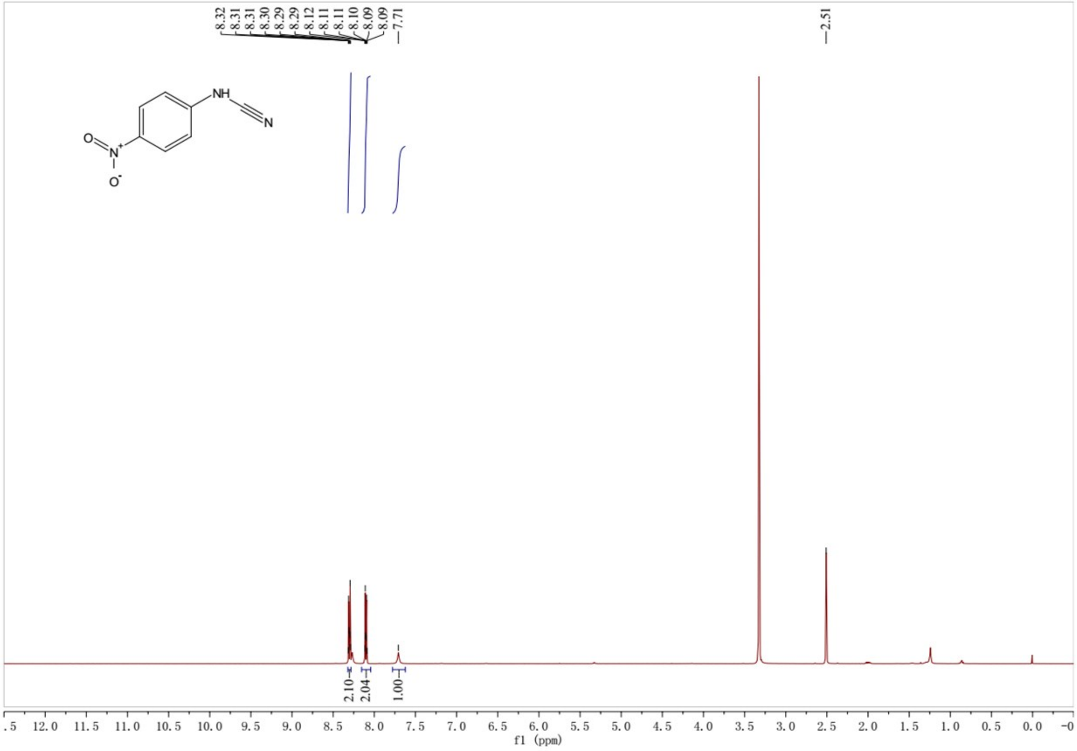

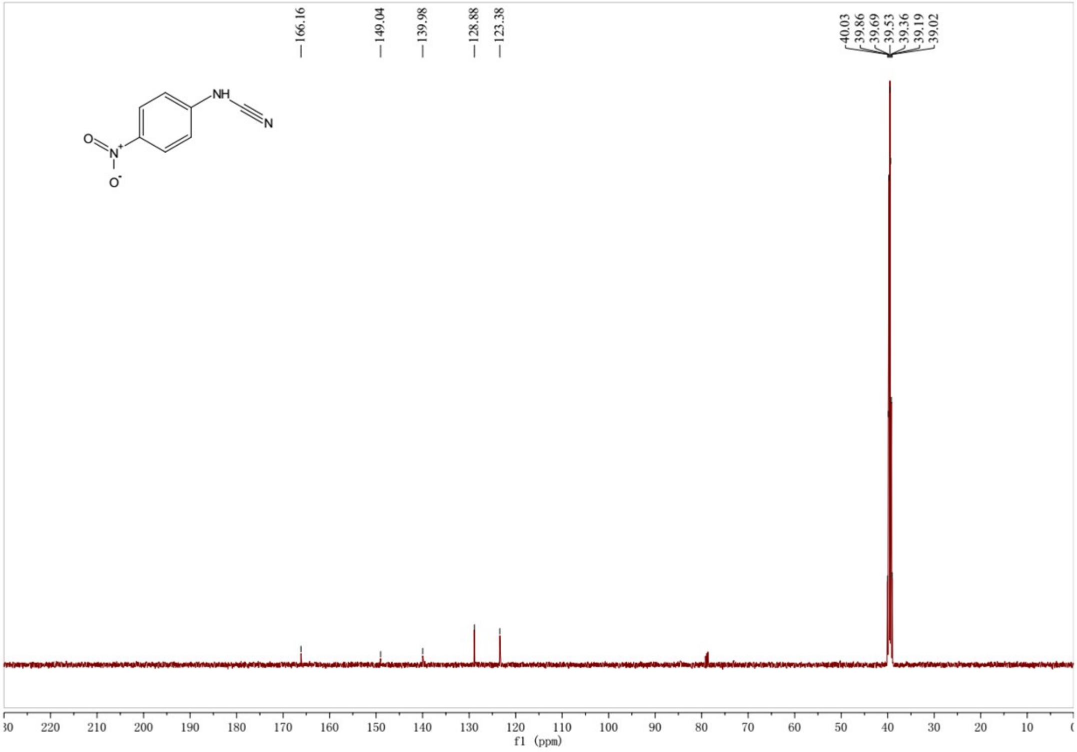

N-(4-Dimethylaminophenyl)cyanamide (2j)

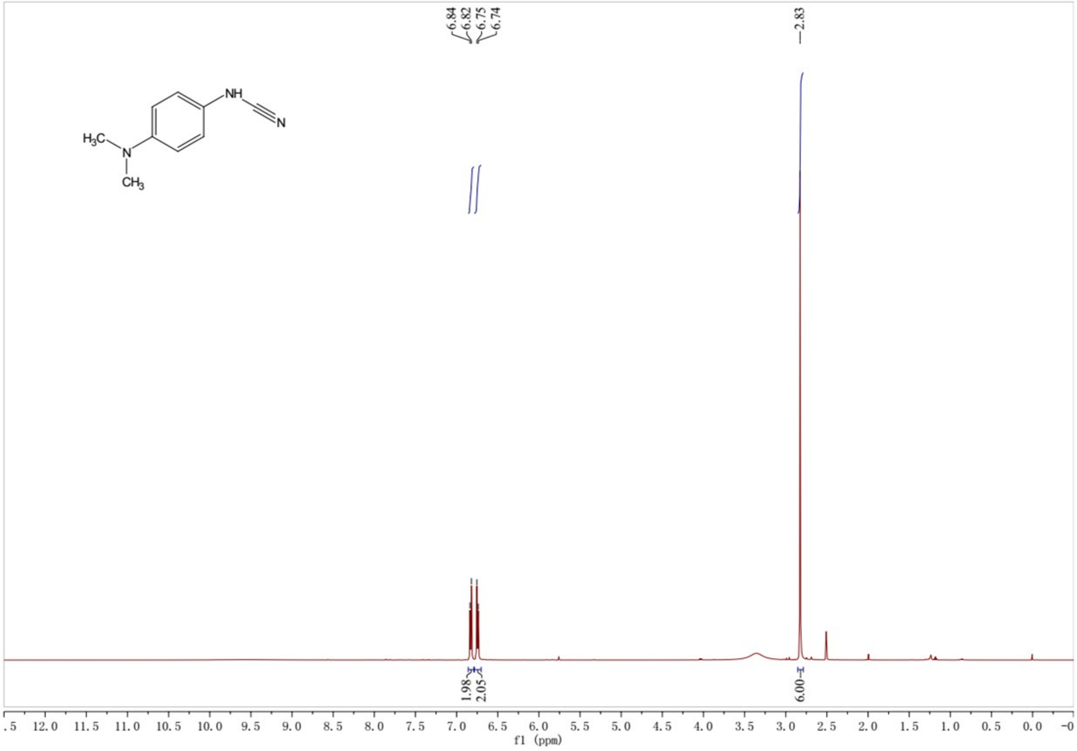

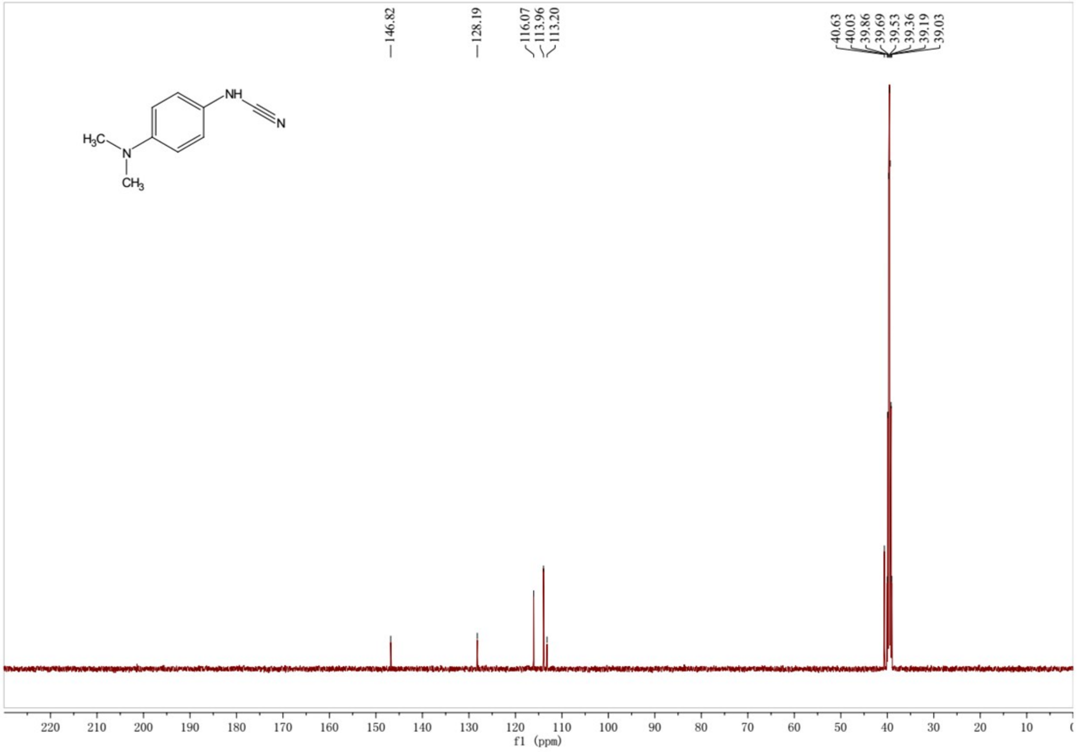

N-(m-Tolyl)cyanamide (2k)

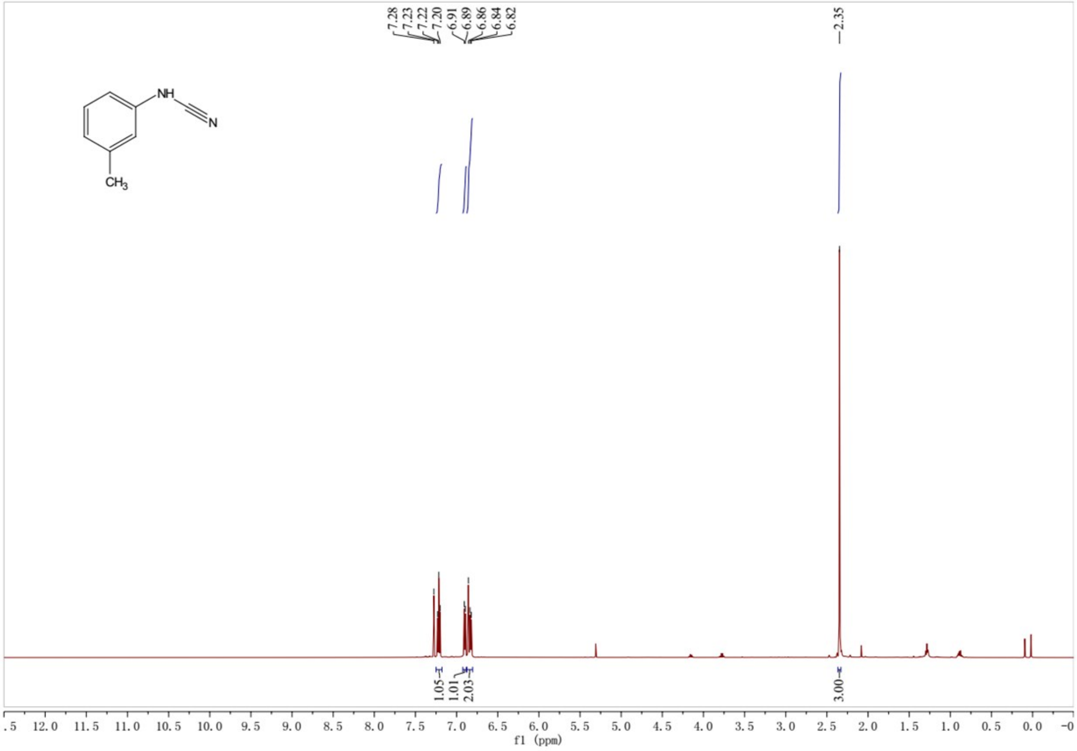

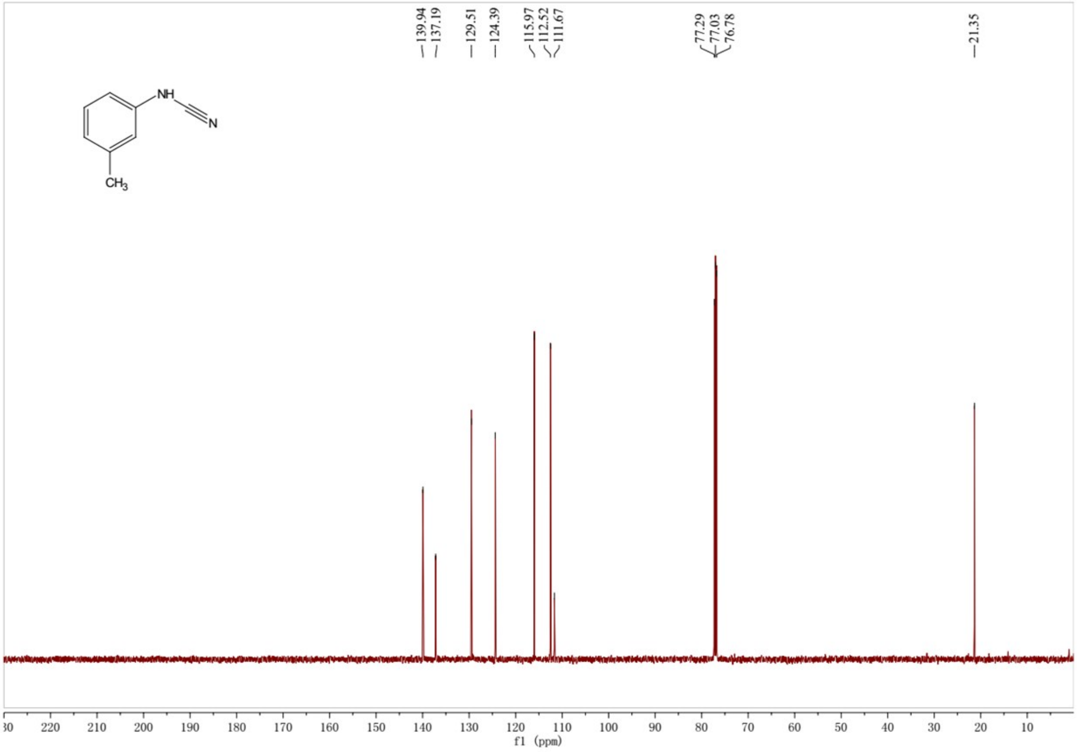

***N*-(3-Bromophenyl)cyanamide (2l)**

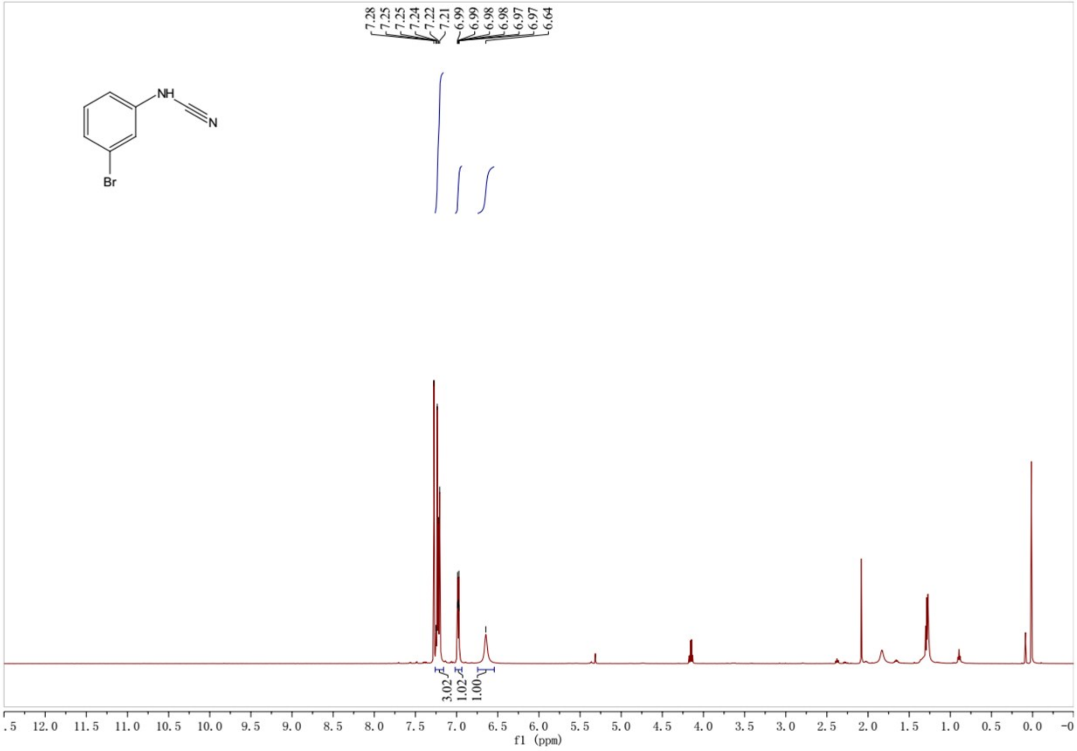

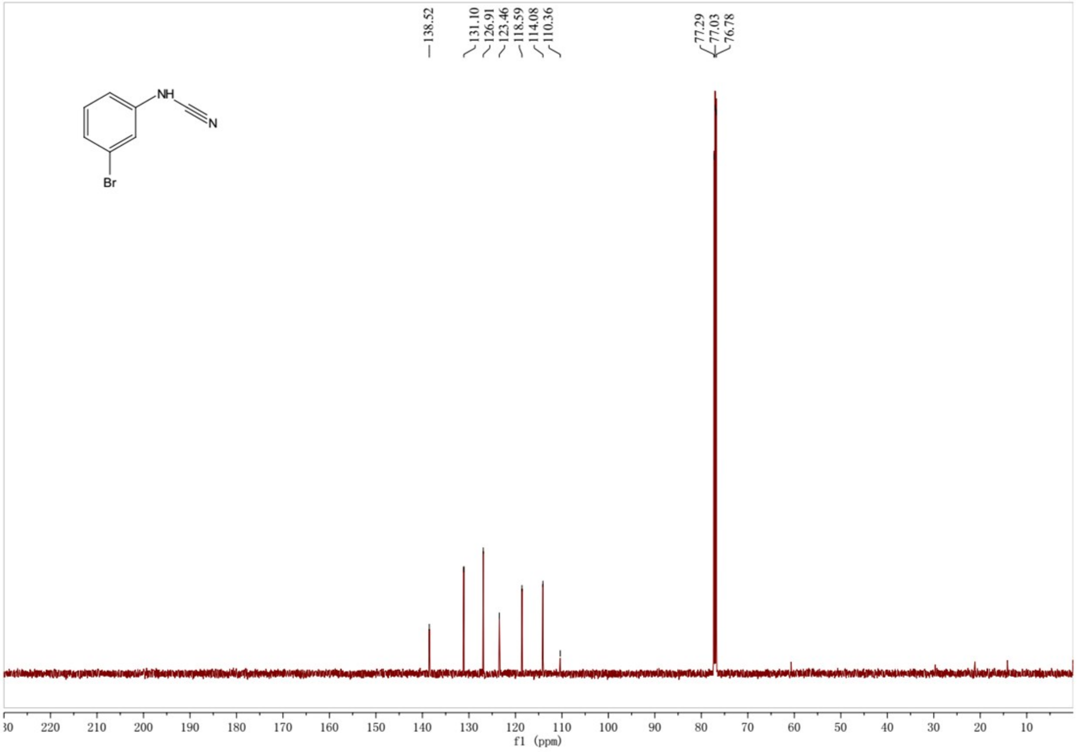

***N*-(3-Trifluoromethylphenyl)cyanamide (2m)**

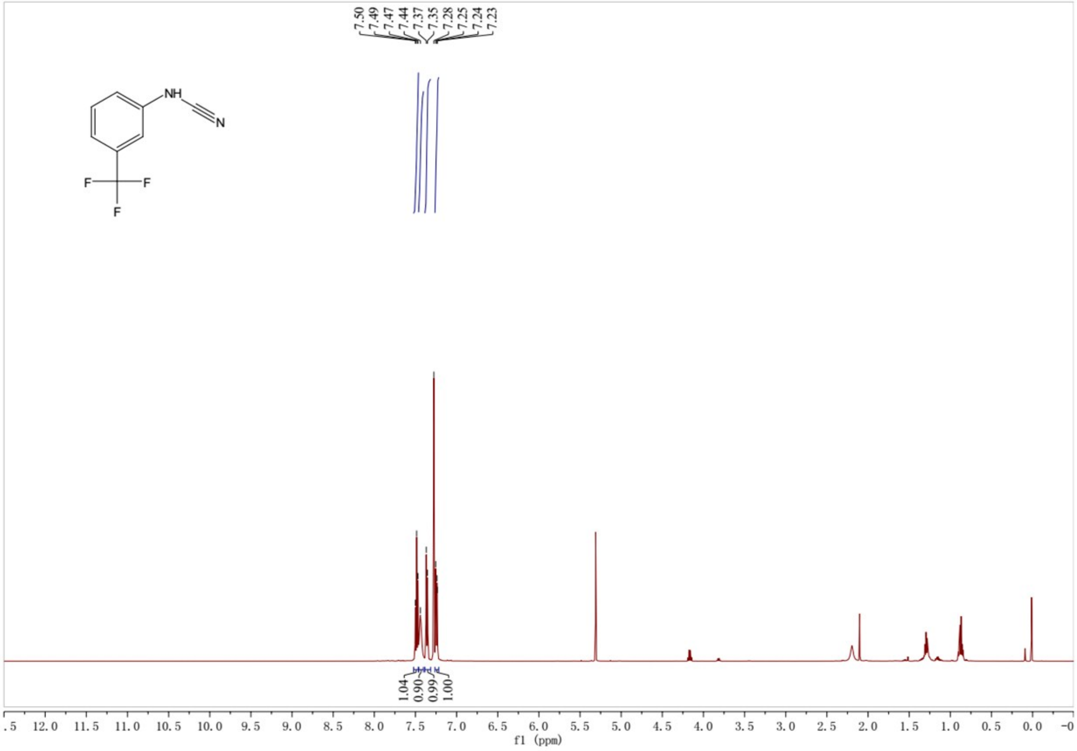

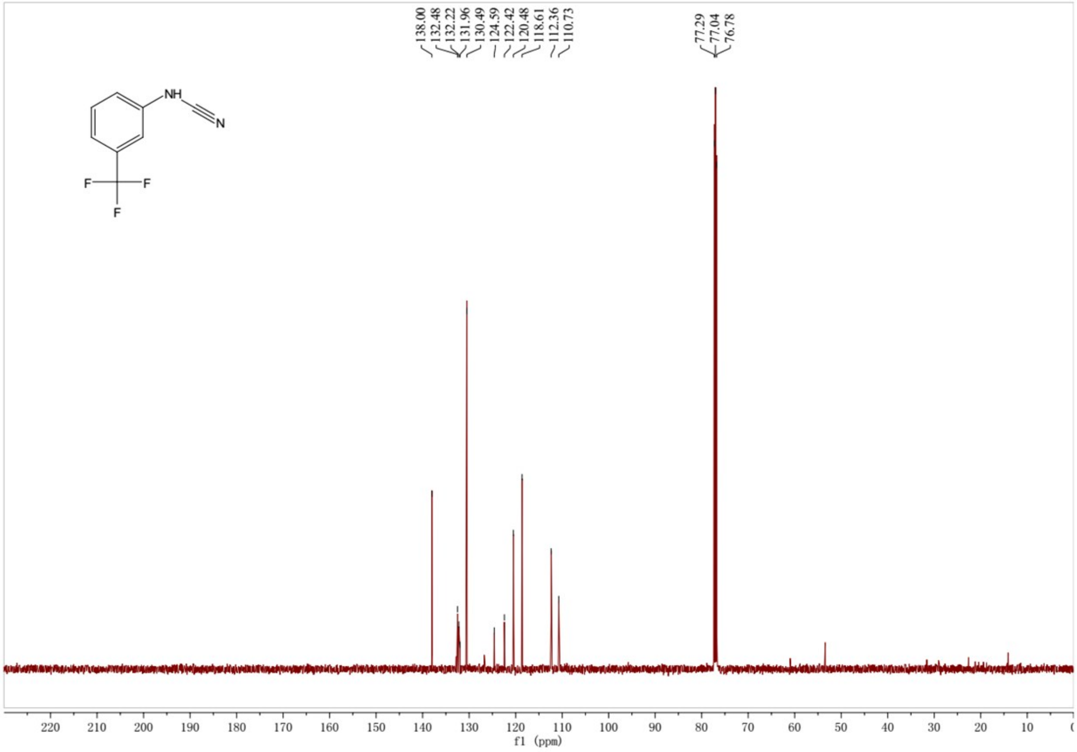

N-(o-Tolyl)cyanamide (2n)

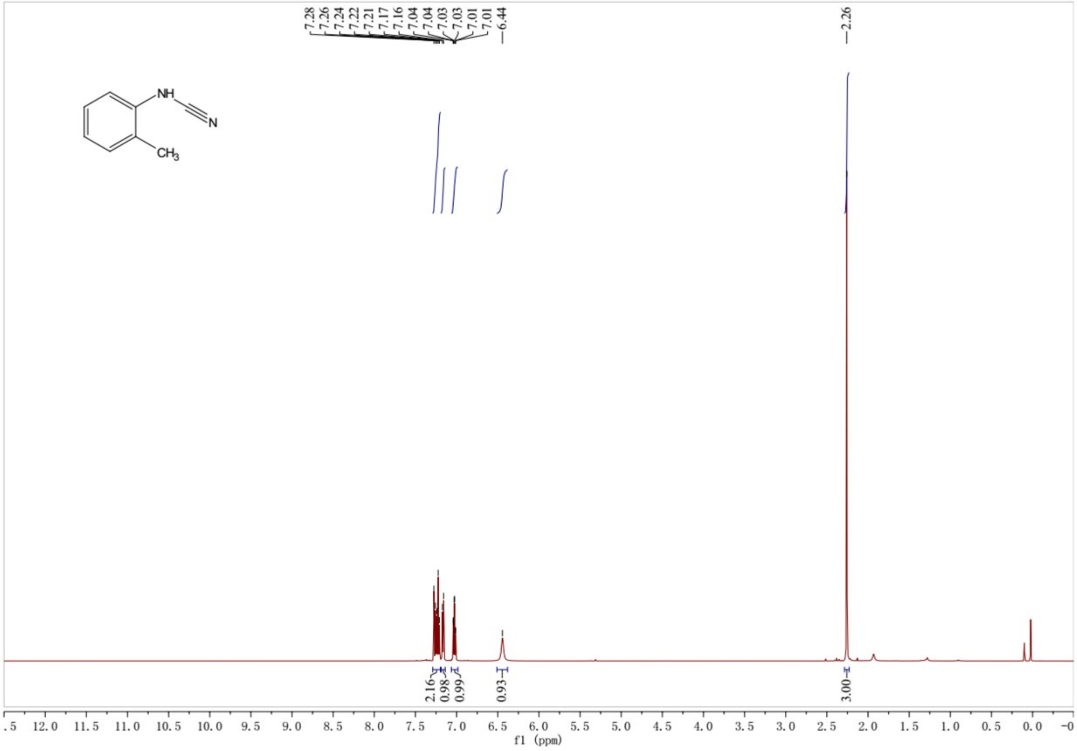

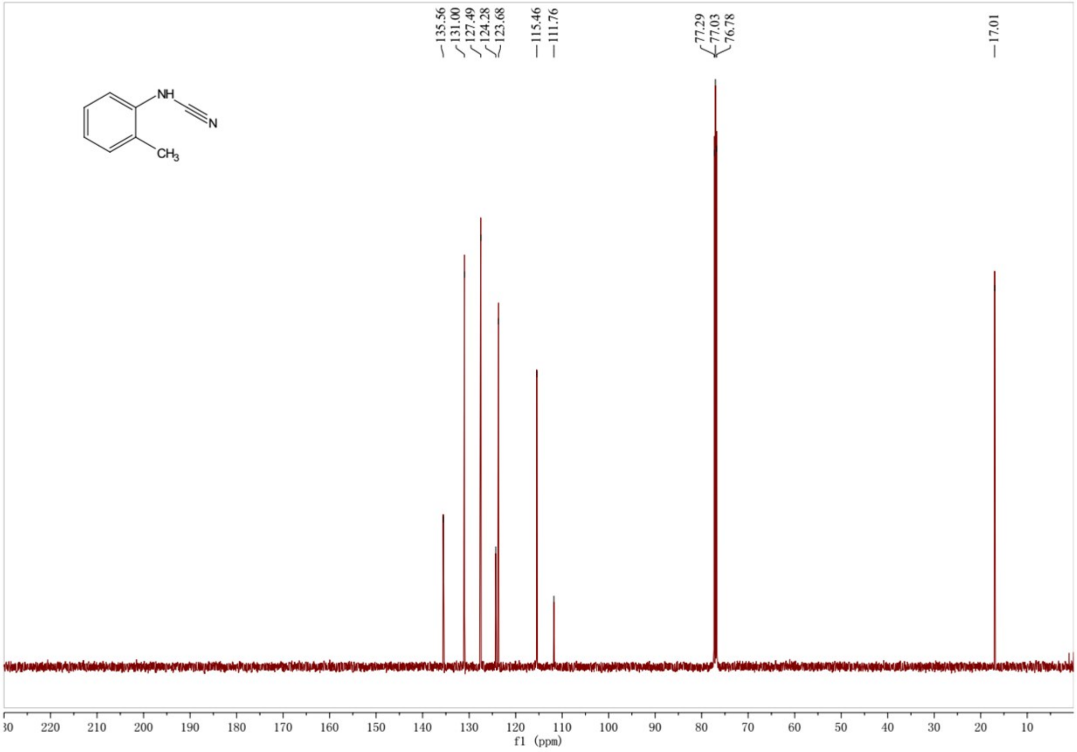

_N_-(2-Bromophenyl)cyanamide (2o)

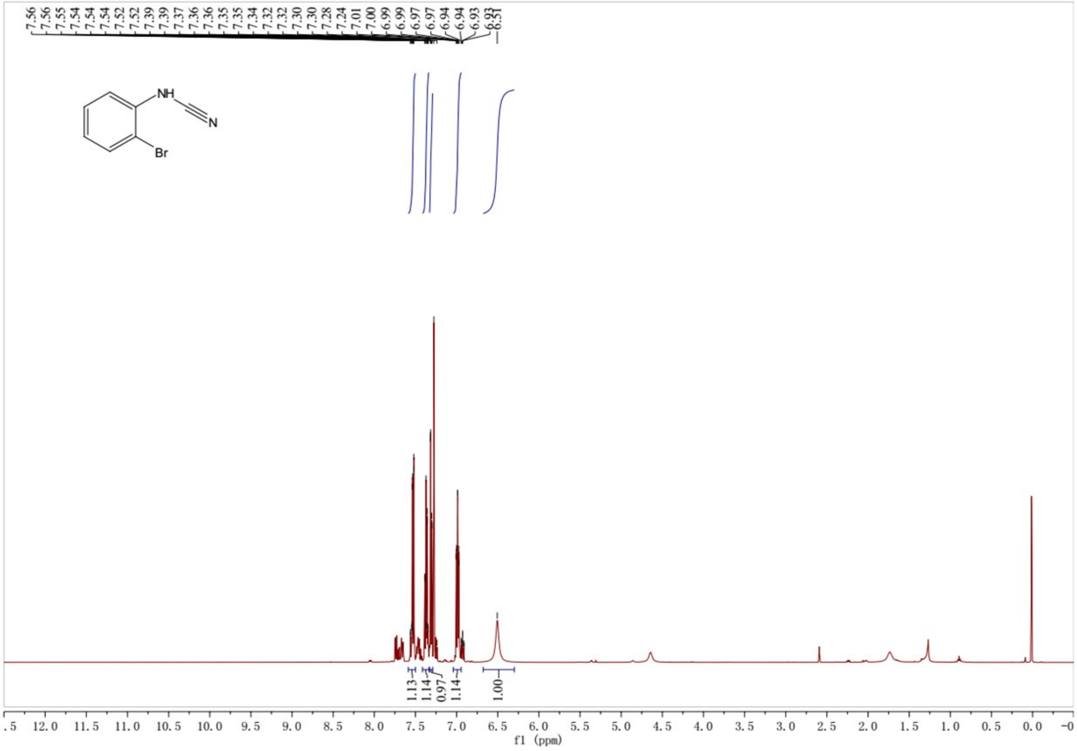

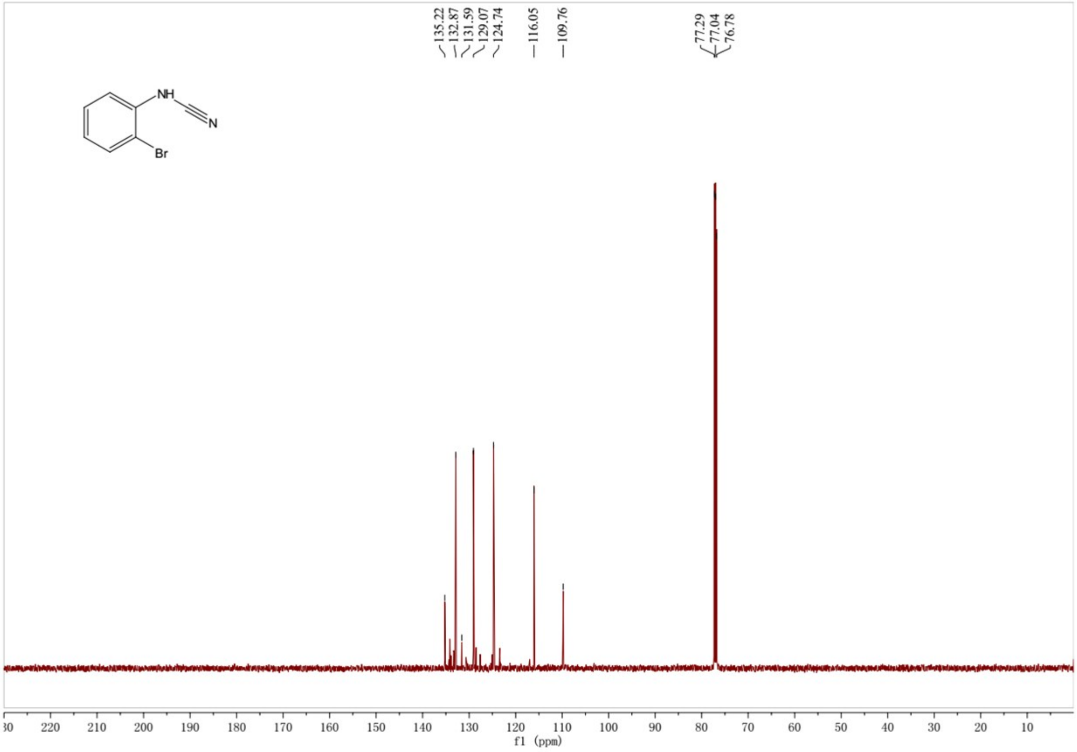

N-(2-Trifluoromethylphenyl)cyanamide (2p)

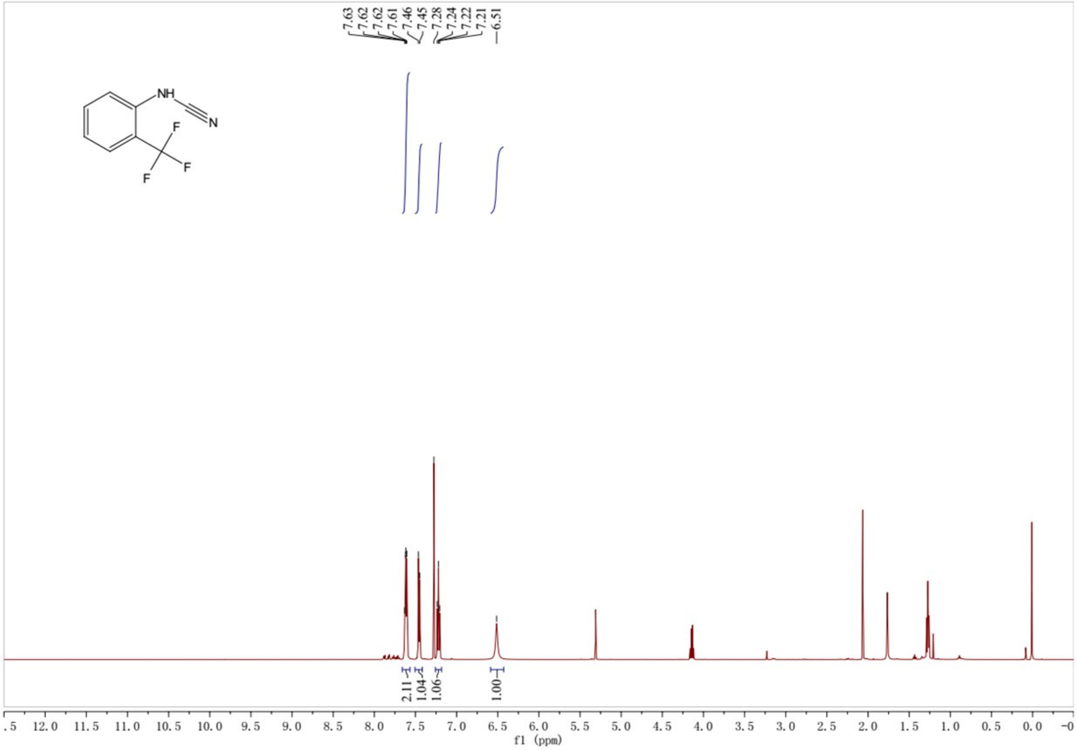

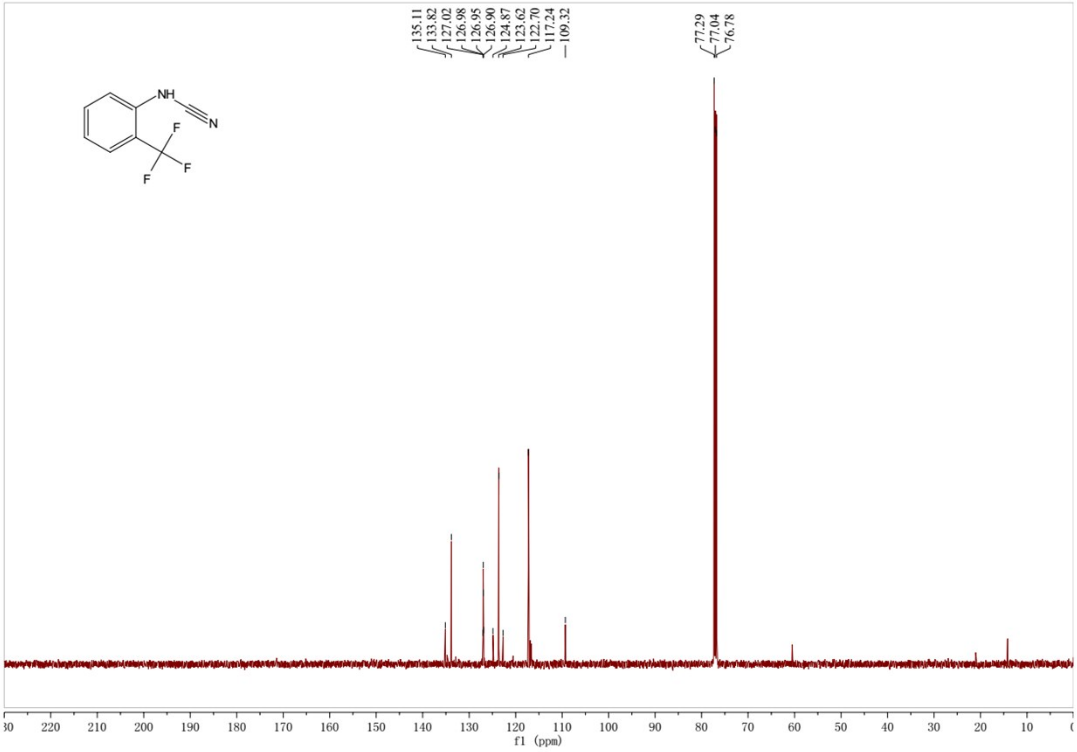

N-(3,4-Dimethoxyphenyl)cyanamide (2q)

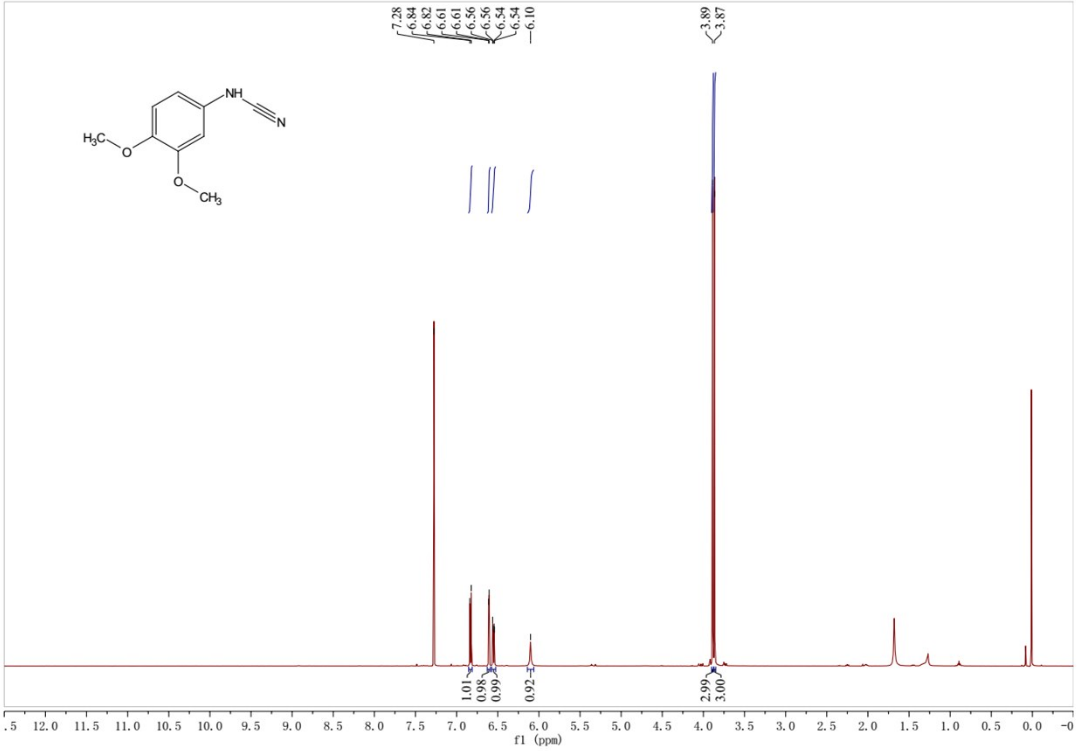

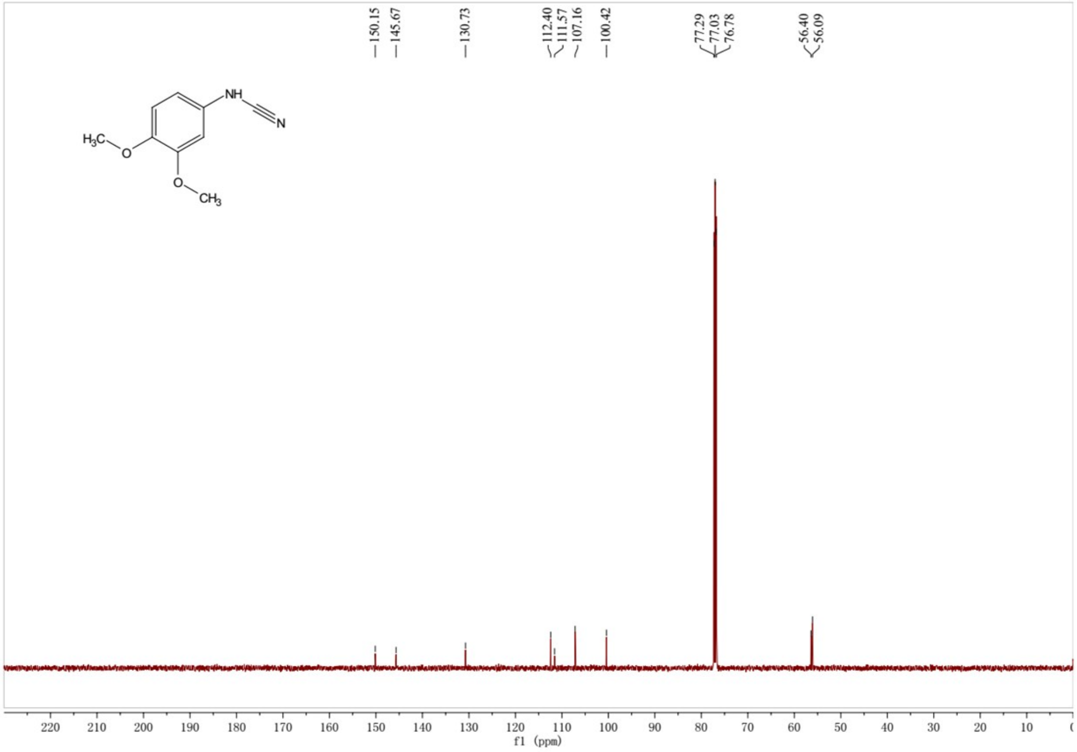

***N*-(Benzo[d][1,3]dioxol-5-yl)cyanamide (2r)**

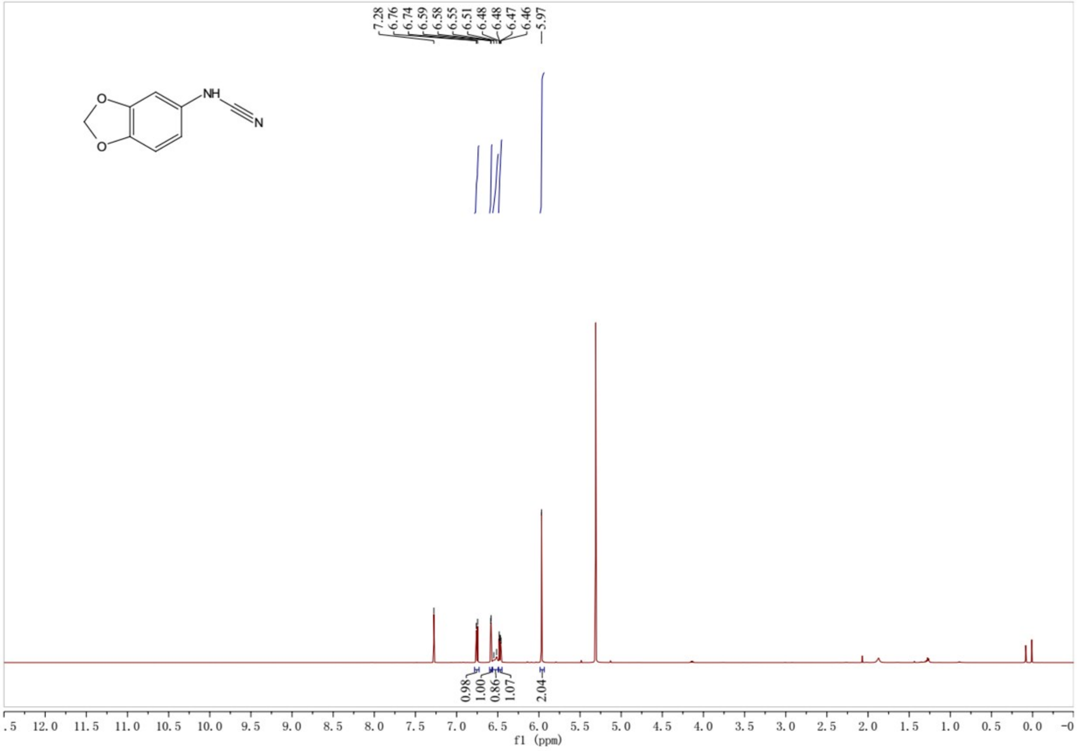

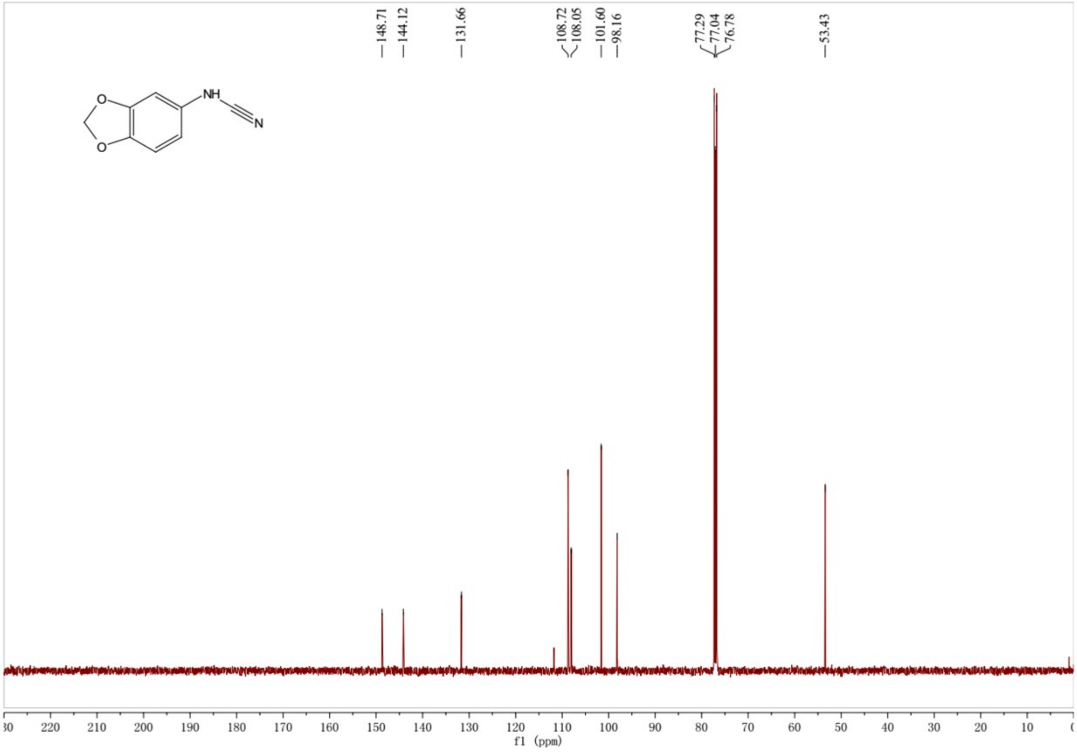

N-(Naphthalen-2-yl)cyanamide (2s)

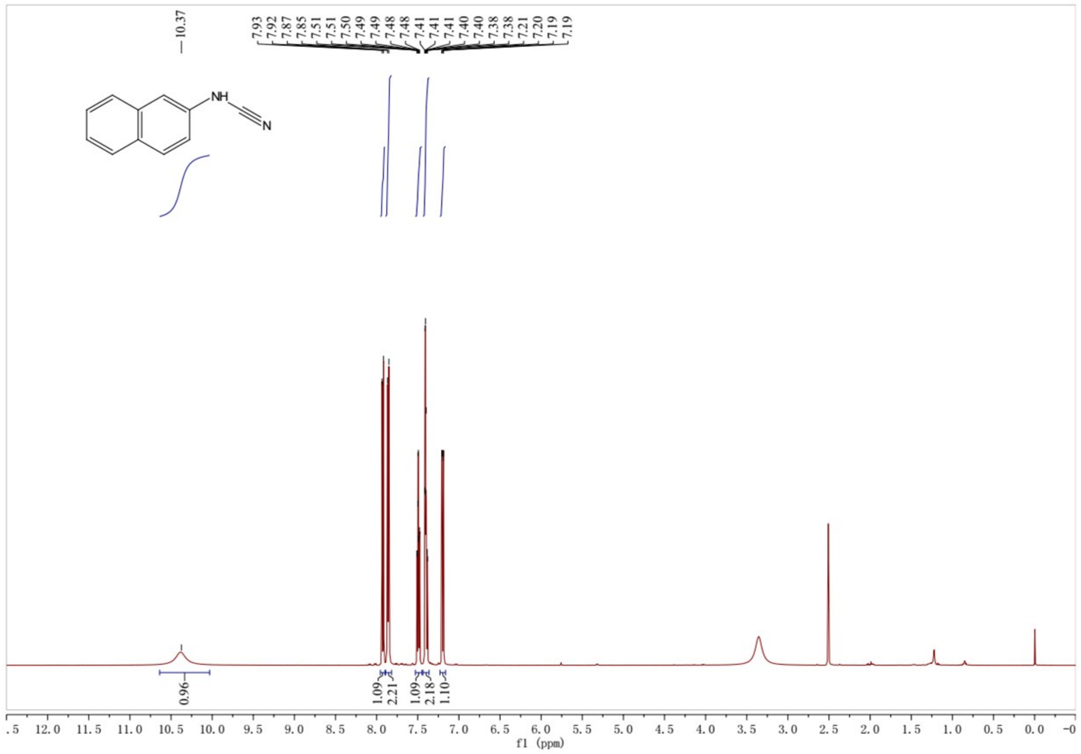

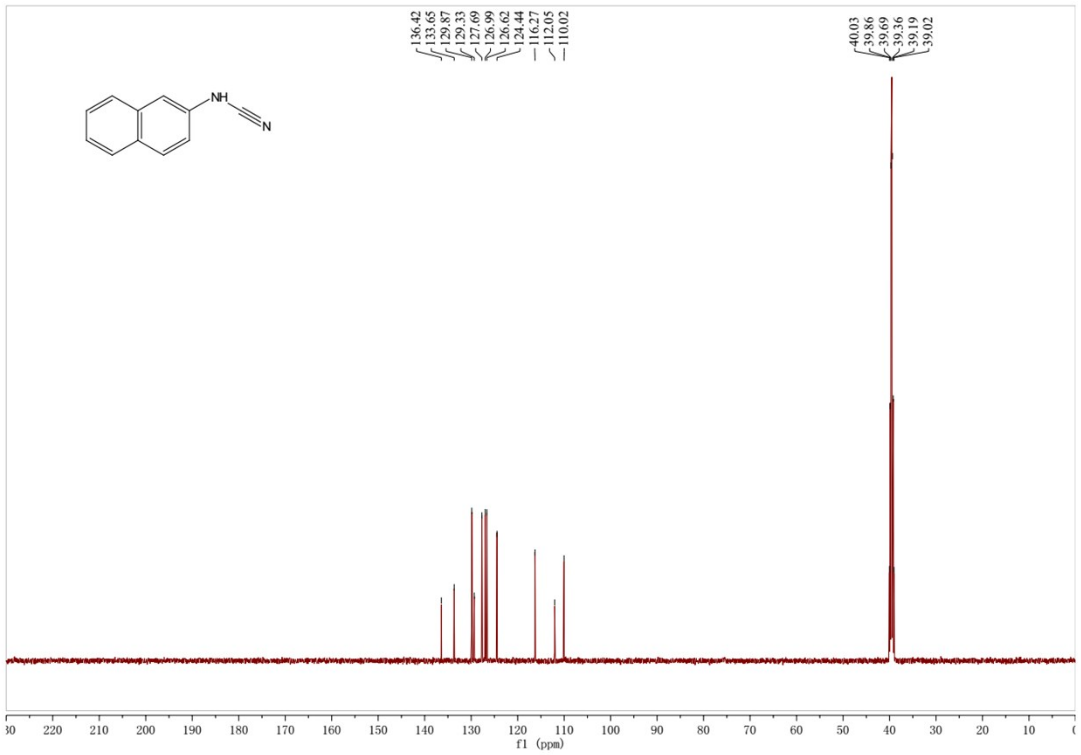

N-(Naphthalen-1-yl)cyanamide (2t)

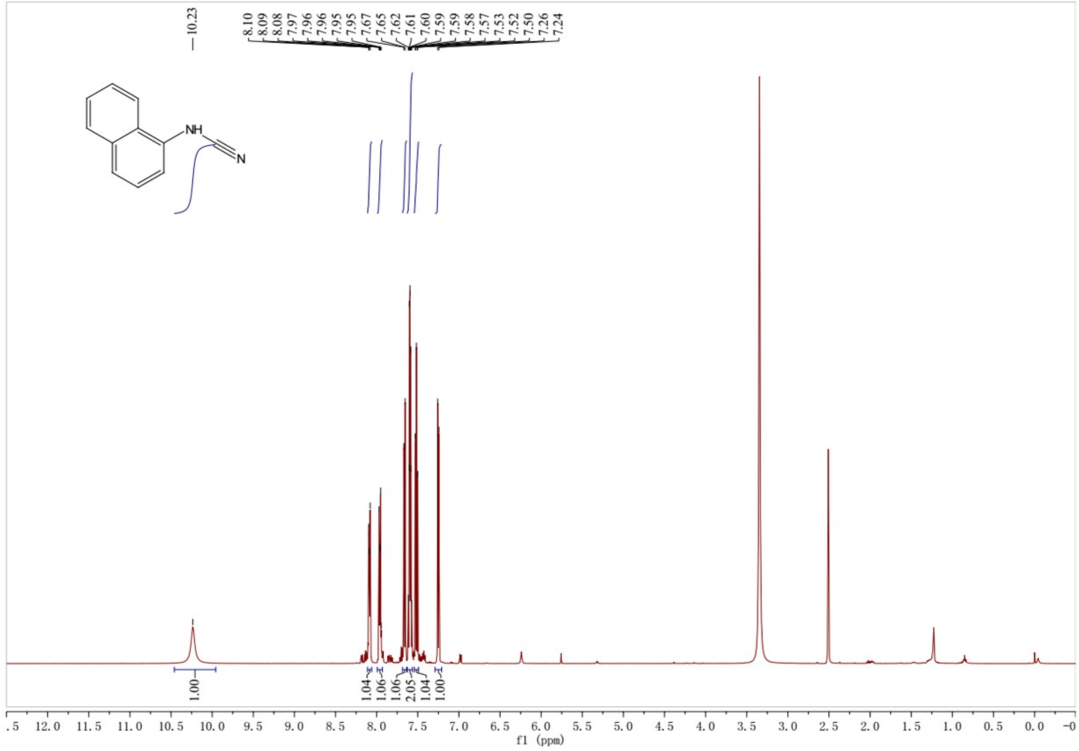

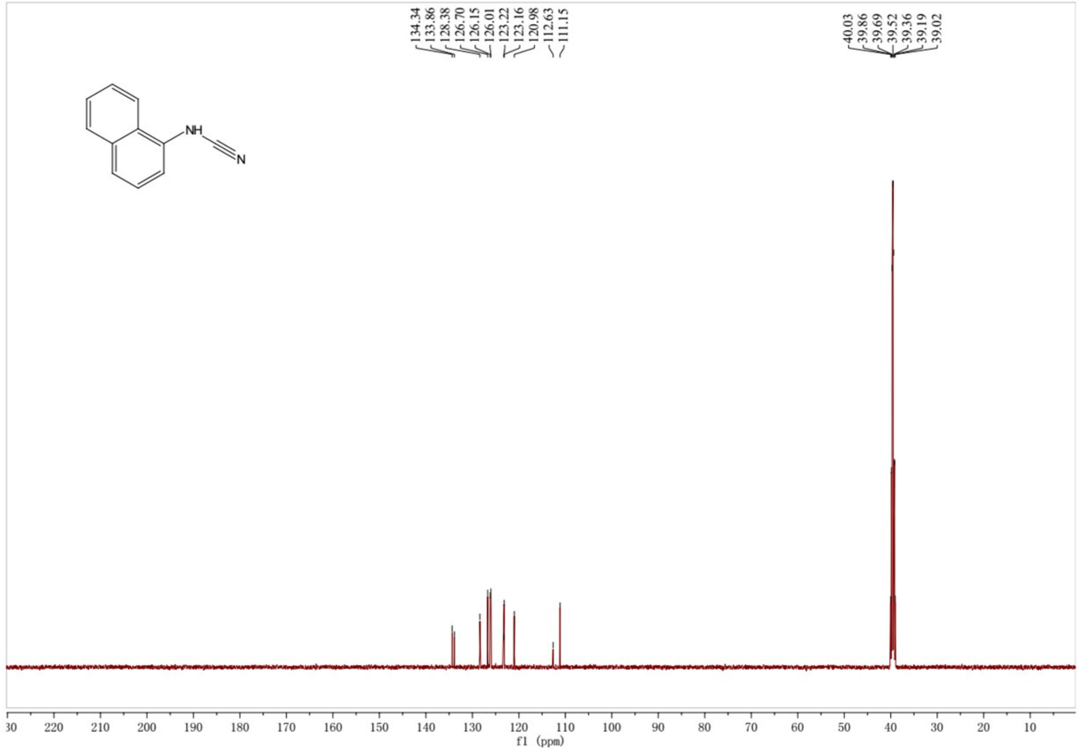

***N*-(1*H*-Indol-5-yl)cyanamide (2u)**

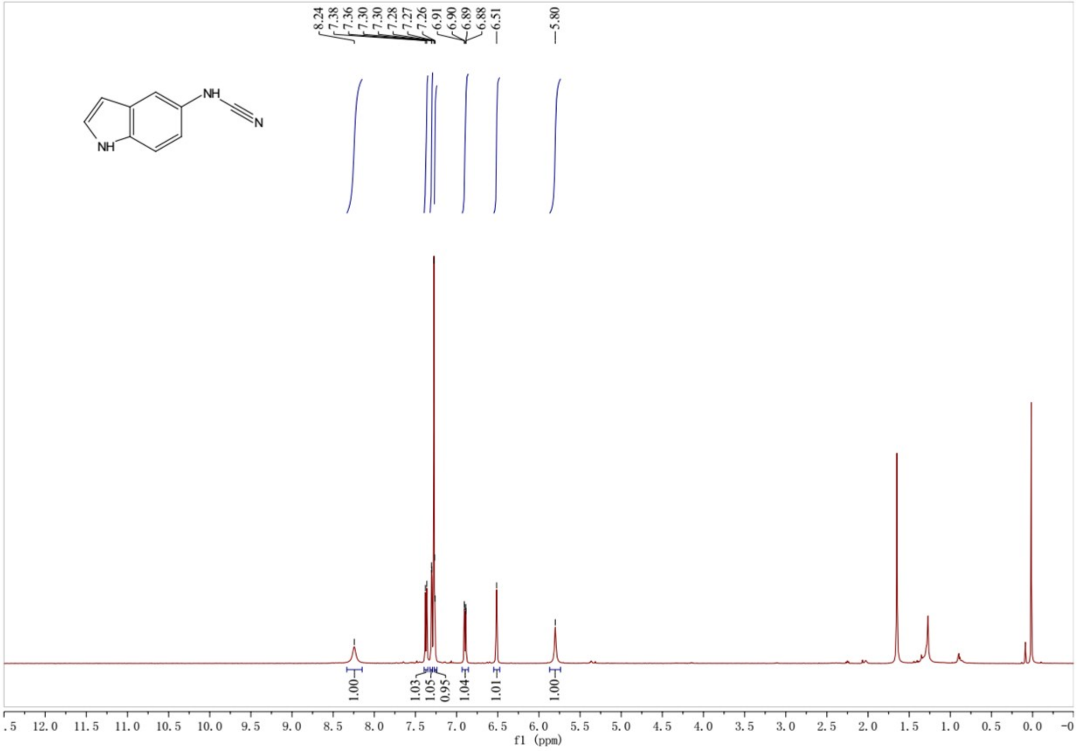

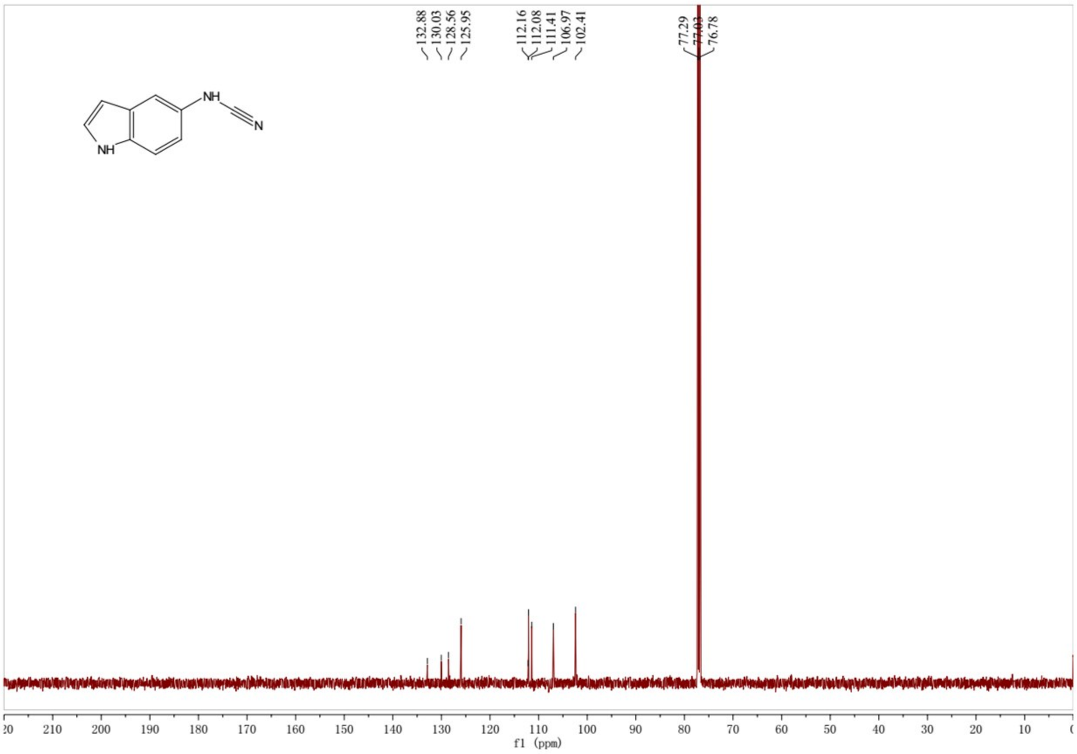

***N*-(Pyridin-2-yl)cyanamide (2v)**

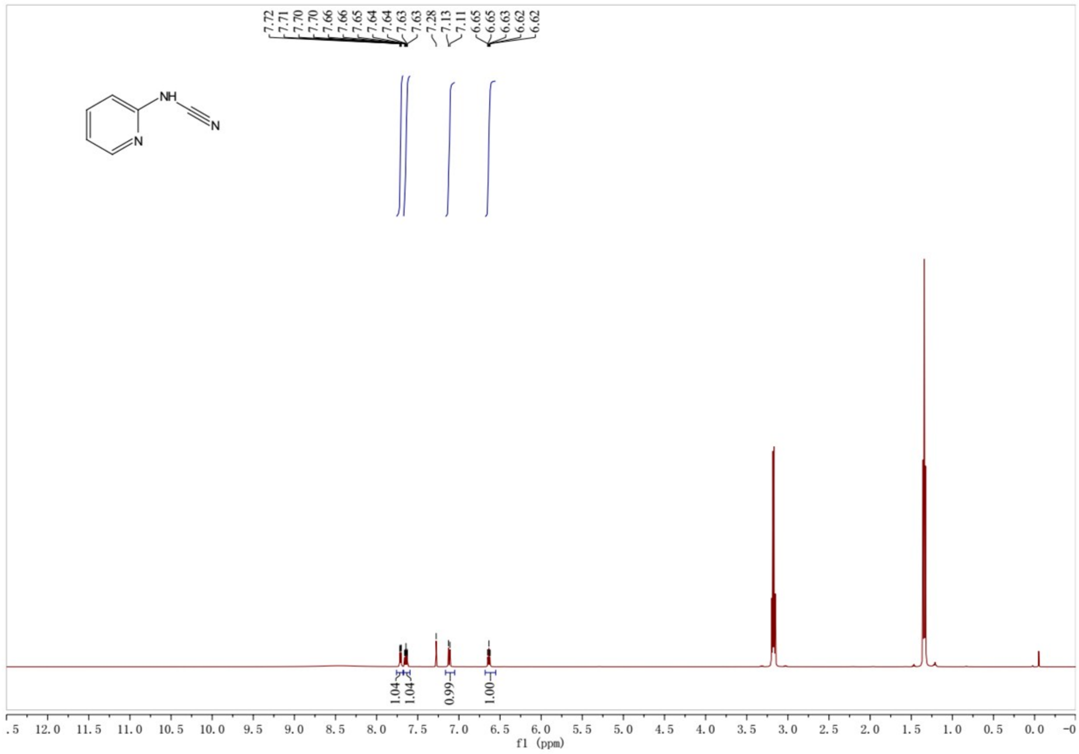

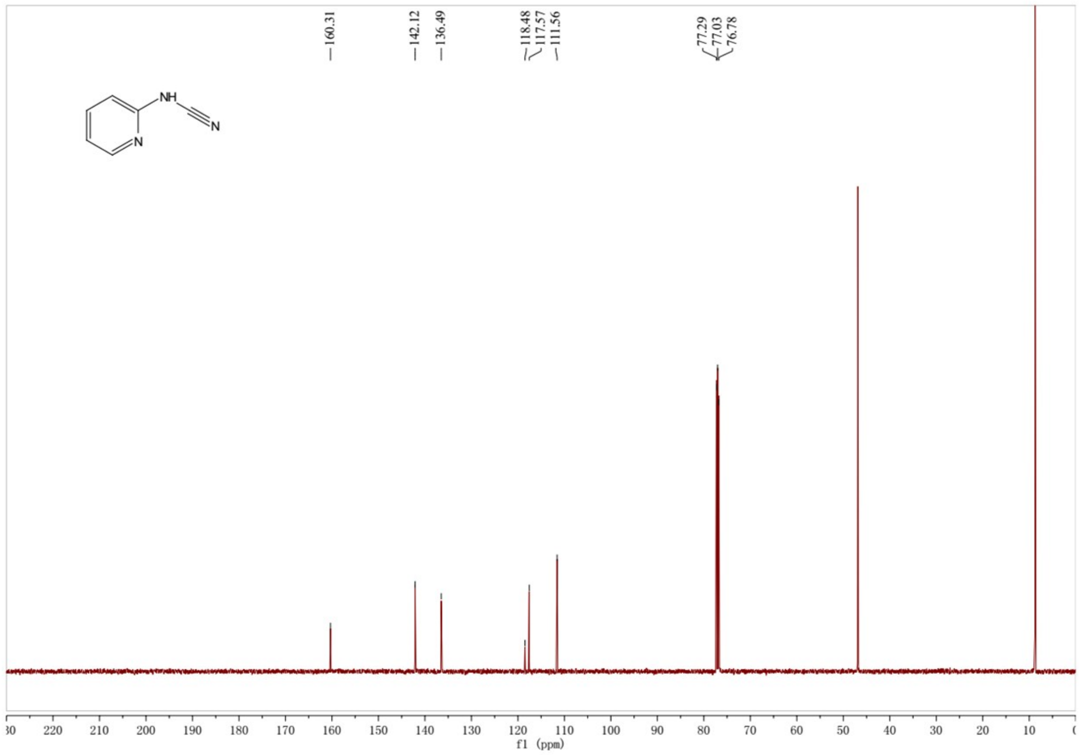

***N*-(Phenylethynyl)cyanamide (2w)**

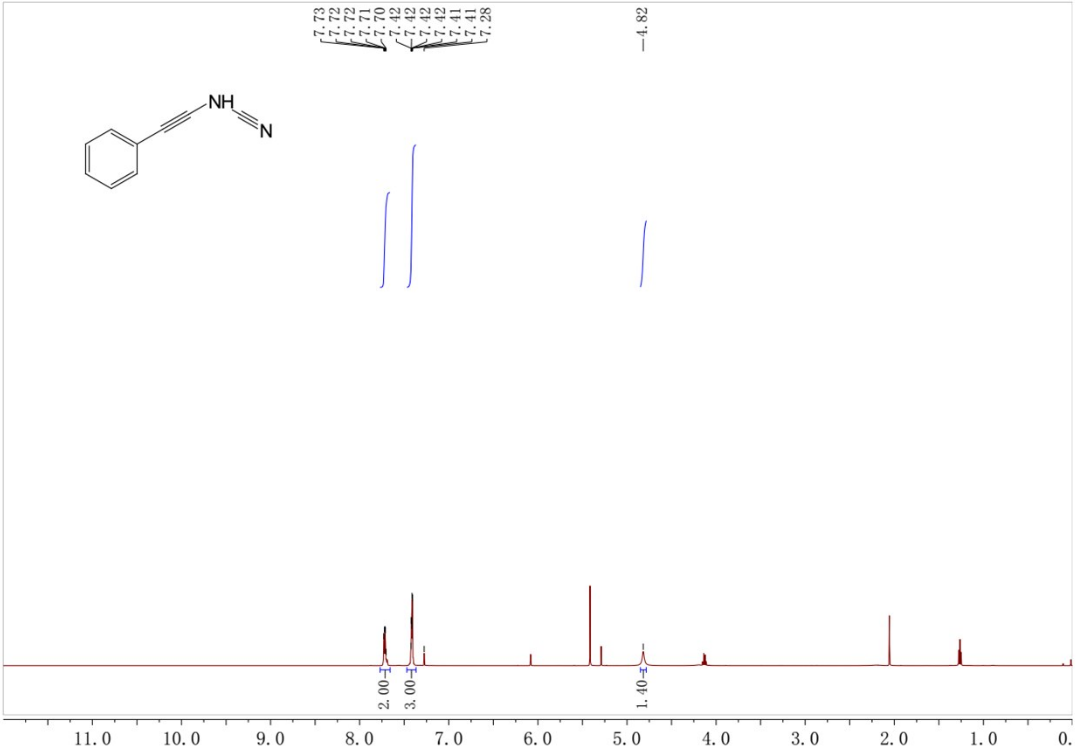

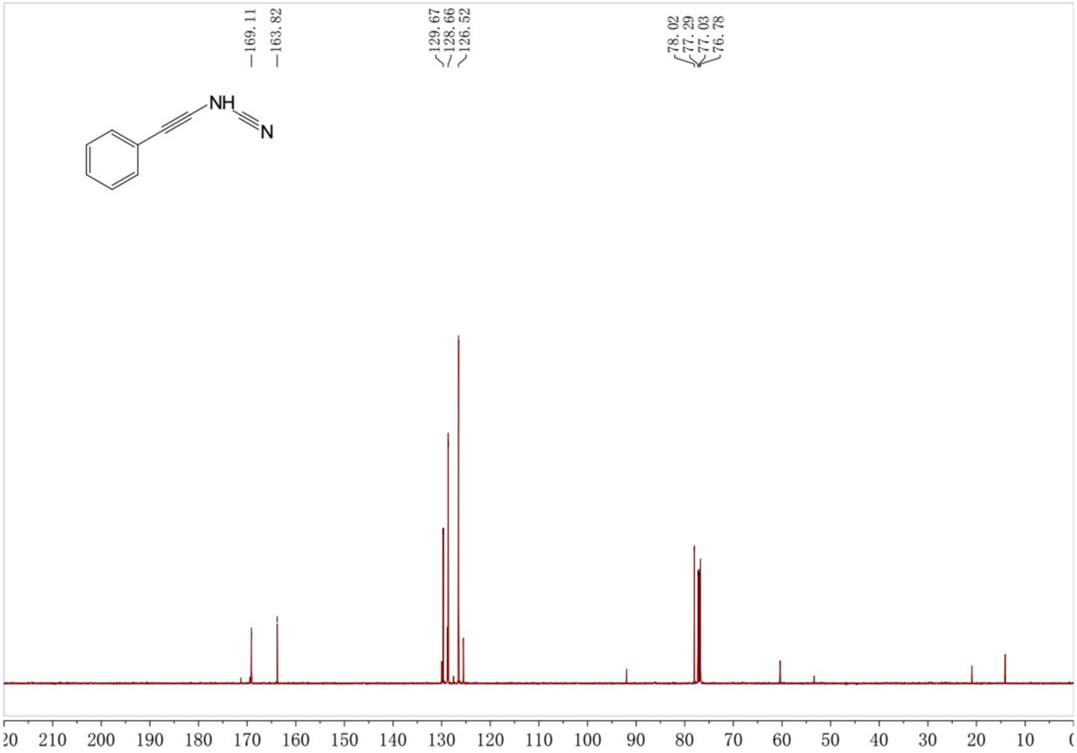

***N*-Benzylcyanamide (2x)**

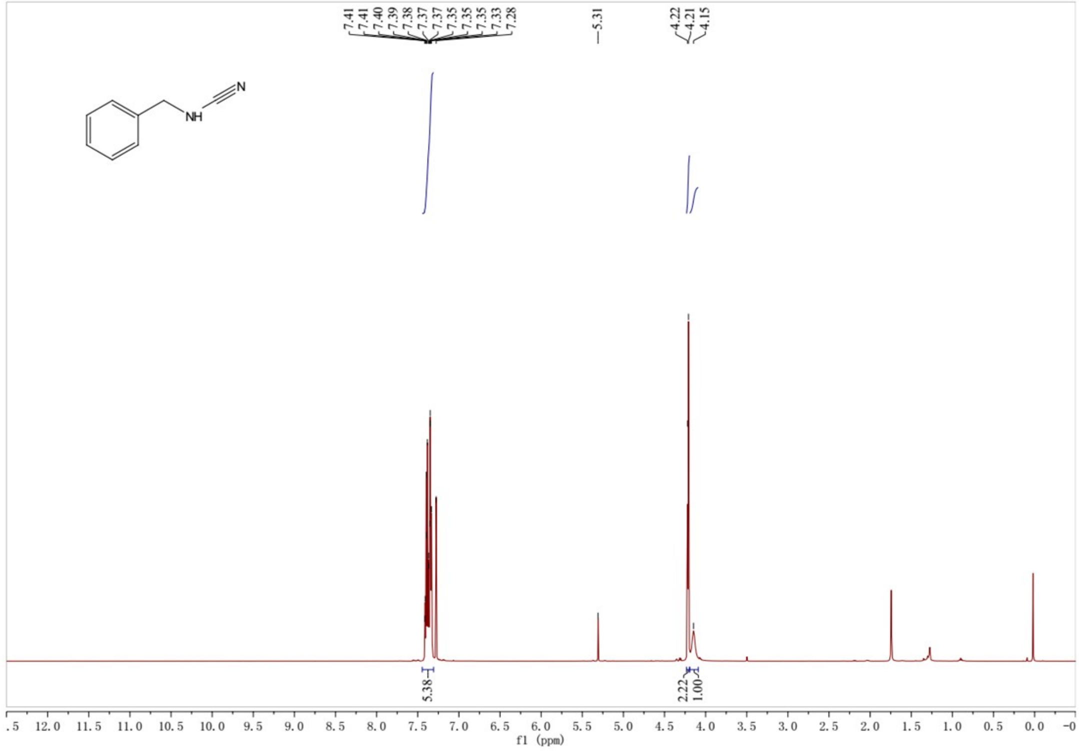

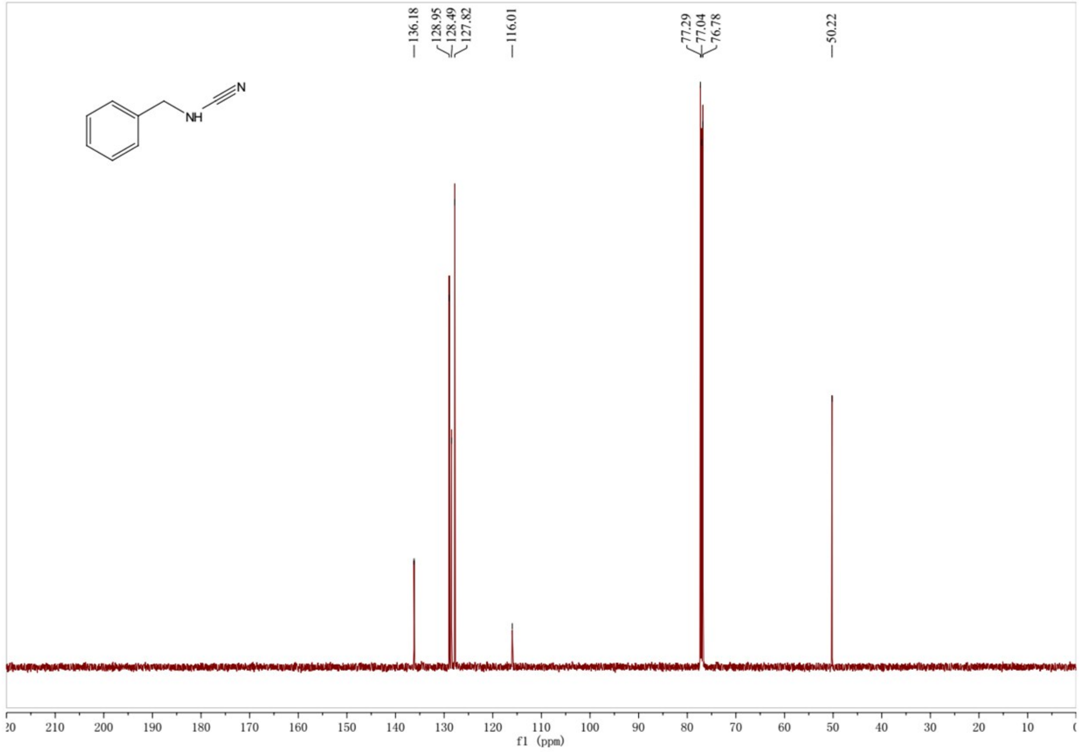

***N*-Phenethylcyanamide (2y)**

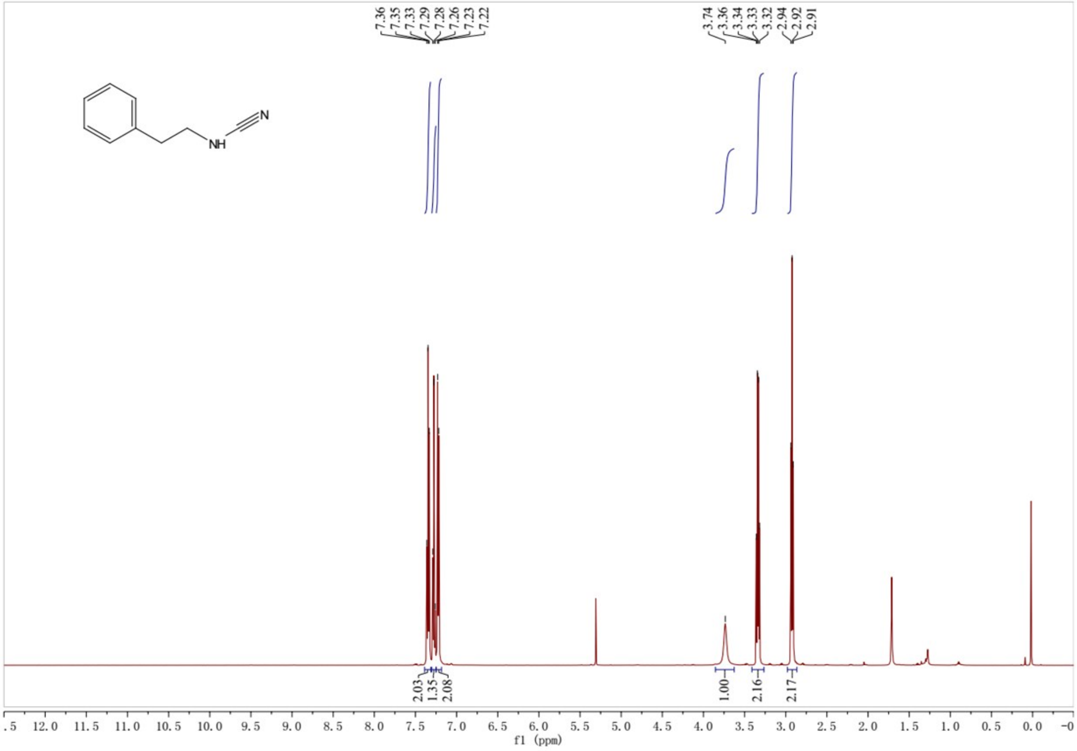

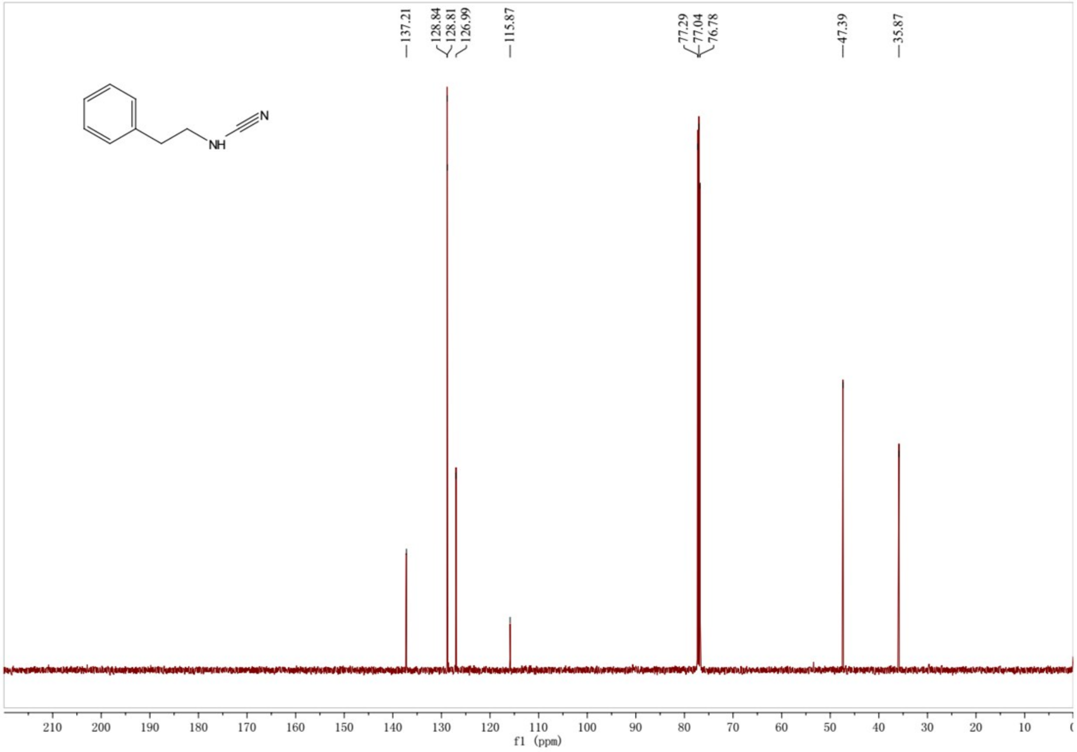

N-(*tert*-Butyl)cyanamide (2z)

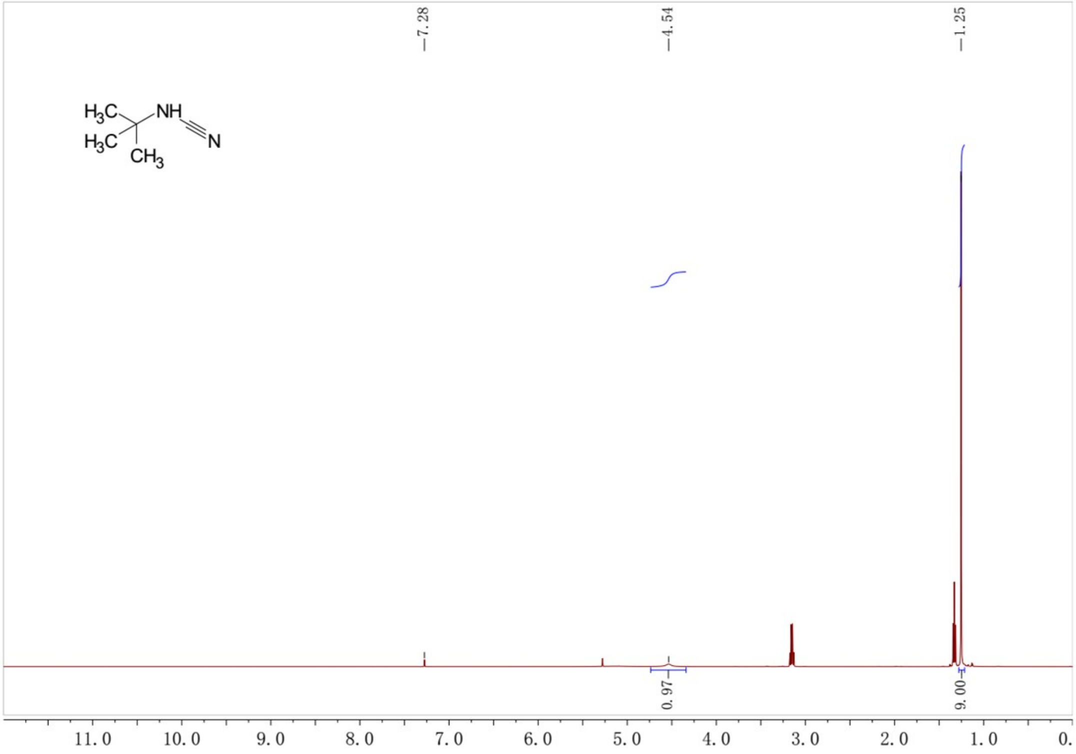

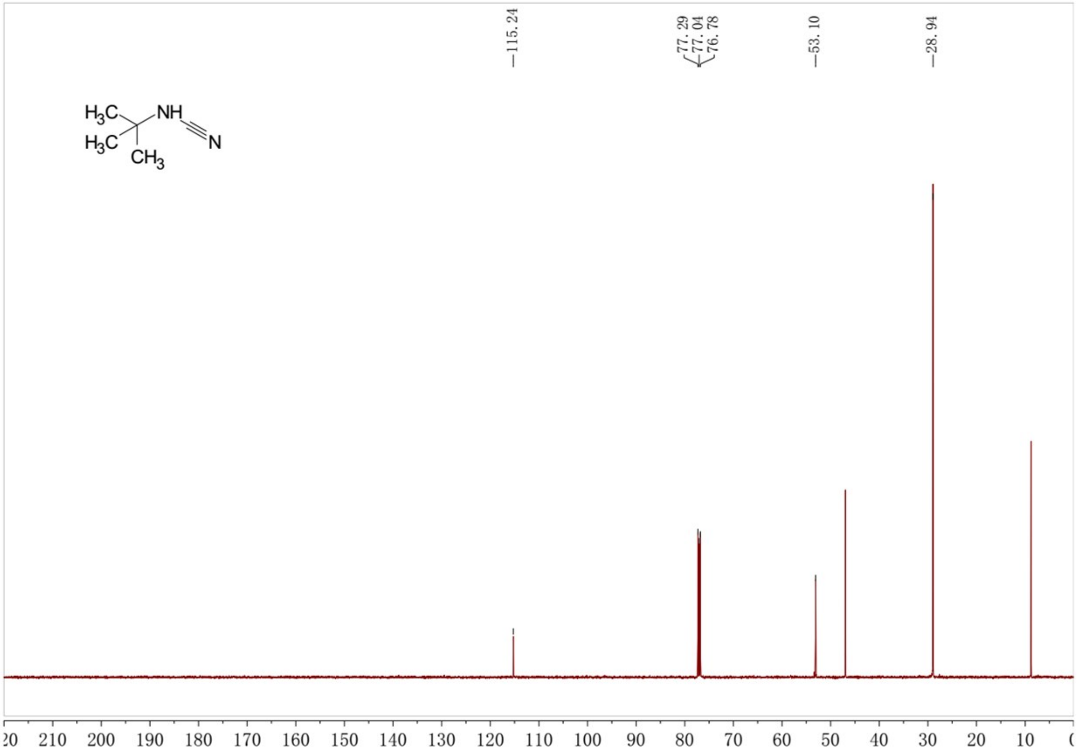

Supplement: RA-010-D0RA02631J-s001 [file RA-010-D0RA02631J-s001.pdf]
